# Supplementary material for: Stimuli-responsive mechanically interlocked polymer wrinkles
Source: Nat Commun. 2024 Jul 9;15:5760. doi: 10.1038/s41467-024-49750-8 (PMC11233622; doi:10.1038/s41467-024-49750-8)
Supplement: Supplementary file 1 — Supplementary Information [file 41467_2024_49750_MOESM1_ESM.pdf]

Supplementary Information for

Stimuli-responsive mechanically interlocked polymer wrinkles

Mengling Yang[12], Shuai Chen[12], Zhaoming Zhang[1], Lin Cheng[1], Jun Zhao[1], Ruixue Bai[1], Wenbin Wang[1], Wenzhe Gao[1], Wei Yu[1], Xuesong Jiang[1*], and Xuzhou Yan[1*]

[1]School of Chemistry and Chemical Engineering, Frontiers Science Center for Transformative Molecules, Shanghai Jiao Tong University, Shanghai 200240, P. R. China.

[2]These authors contributed equally: Mengling Yang, Shuai Chen.

[*]Corresponding authors. E-mails: ponygle@sjtu.edu.cn, xzyan@sjtu.edu.cn

1. Materials

All reagents were commercially available and used as supplied without further purification. Deuterated solvents were purchased from Cambridge Isotope Laboratory (Andover, MA).

2. Syntheses of the monomers for the MINs

Synthesis of the host compound of [2]rotaxane

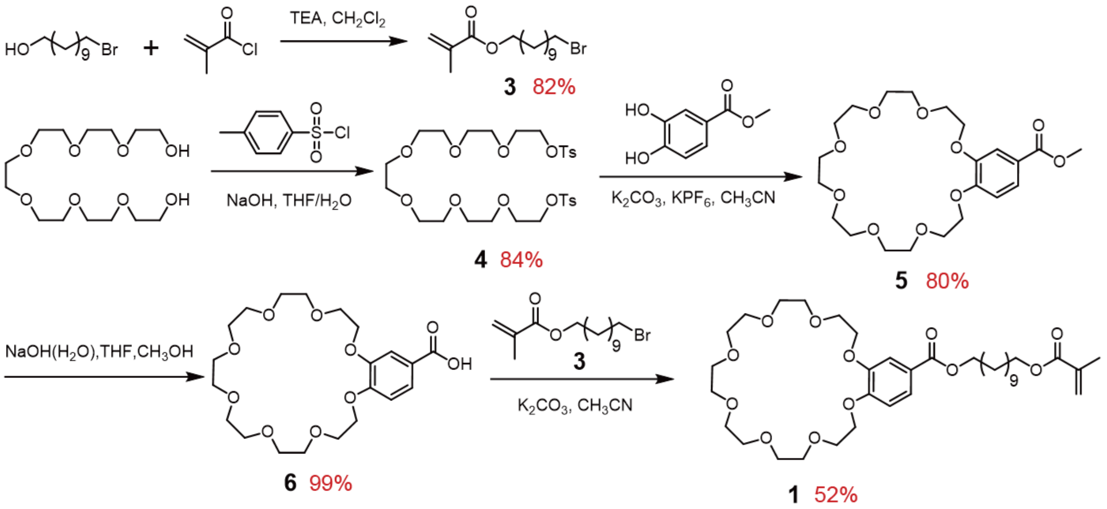

Synthesis of the guest compound of [2]rotaxane

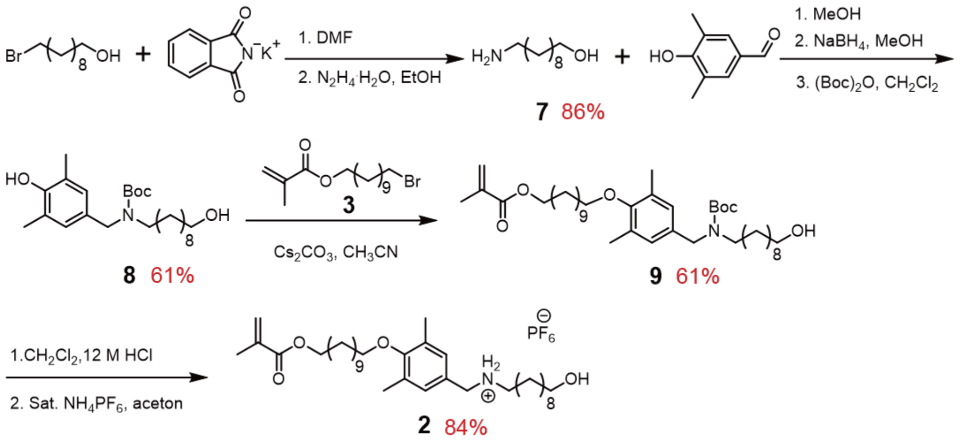

Synthesis of the [2]rotaxane

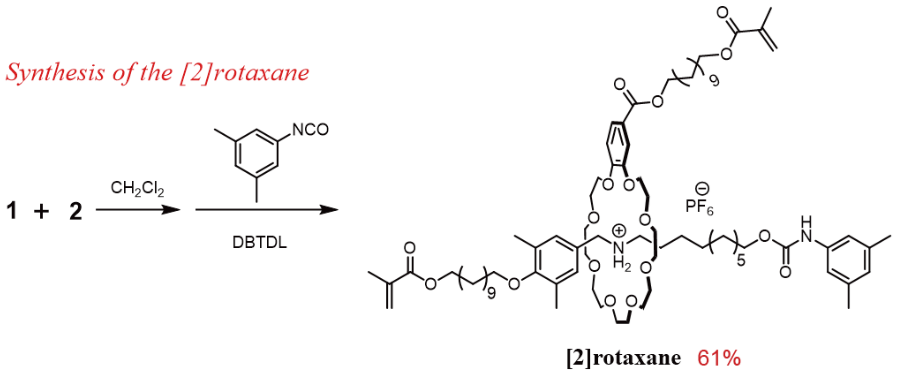

Supplementary Fig. 1 Synthetic route to the [2]rotaxane.

Synthesis of the NB-UPy

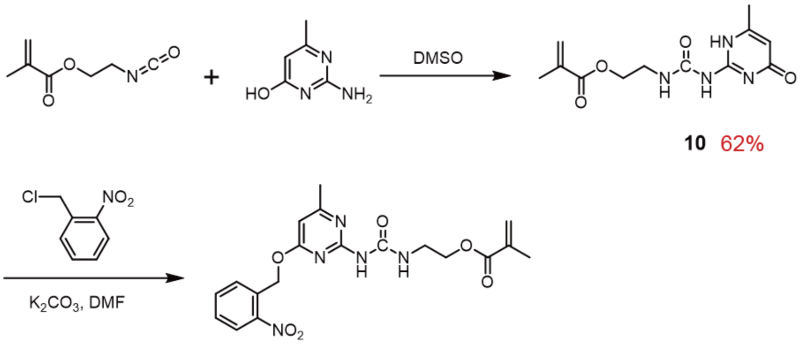

Supplementary Fig. 2 Synthetic route to the NB-UPy.

Synthesis of 11-bromoundecyl methacrylate (3)

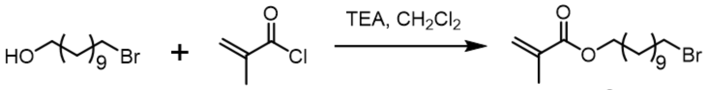

Triethylamine (12.09 g, 119.43 mmol) and 11-bromo-1-undecanol (20.00 g, 79.62 mmol) were mixed in dichloromethane (120 mL) in a flask. Then, a solution of methacryloyl chloride (12.48 g, 119.43 mmol) was added drop by drop over 1 hour at 0 °C. After the addition, the reaction was stirred overnight at room temperature and then filtered to remove the precipitate. The filtrate was purified by gel chromatography (petroleum ether/ethyl acetate, 10:1 *v*/*v*) to afford compound 3 as a colorless liquid (20.80 g, 82%). The ^{1}H NMR spectrum of compound 3 is shown in Supplementary Fig. 3. ^{1}H NMR ($CDCl_3$, room temperature, 400 MHz) δ (ppm): 6.08–6.09 (m, 1H), 5.53–5.55 (m, 1H), 4.13 (t, J = 6.7 Hz, 2H), 3.52 (t, J = 6.8 Hz, 1H), 3.40 (t, J = 6.9 Hz, 1H), 1.94 (t, J = 1.3 Hz, 3H), 1.89–1.60 (m, 5H), 1.47–1.23 (m, 15H). The ^{13}C NMR spectrum of compound 3 is shown in Supplementary Fig. 4. ^{13}C NMR ($CDCl_3$, room temperature, 400 MHz) δ (ppm): 167.55, 136.56, 125.13, 64.82, 45.17, 34.02, 32.83, 29.45, 29.42, 29.39, 29.22, 28.87, 28.75, 28.61, 28.17, 26.88, 25.97. HRESIMS is shown in Supplementary Fig. 5: *m*/*z* calcd for $C_{15}H_{27}BrO_2$, 341.1087 $[M + Na]^+$; found 341.1086 $[M + Na]^+$.

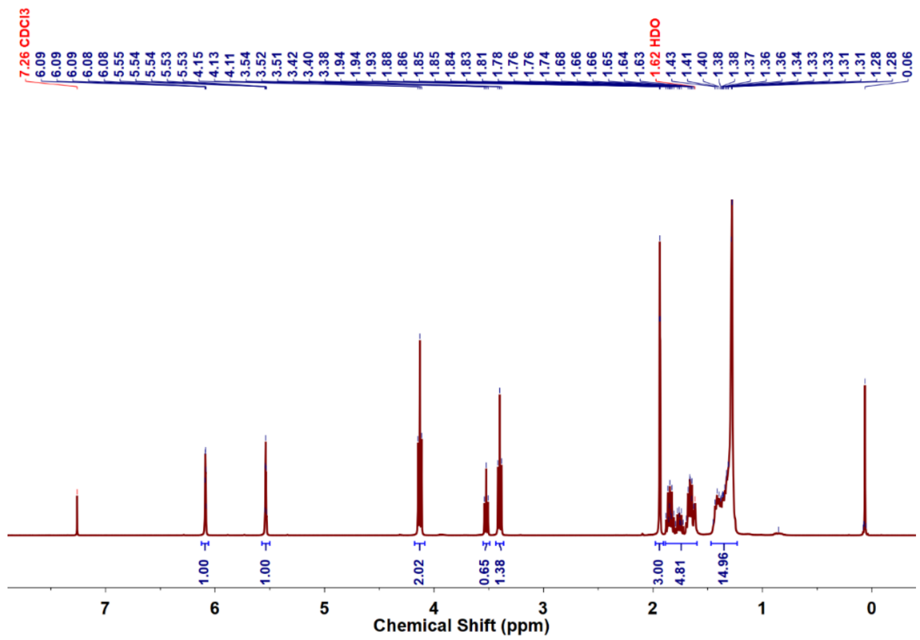

Supplementary Fig. 3 ^{1}H NMR spectrum ($CDCl_3$, room temperature, 400 MHz) of compound 3.

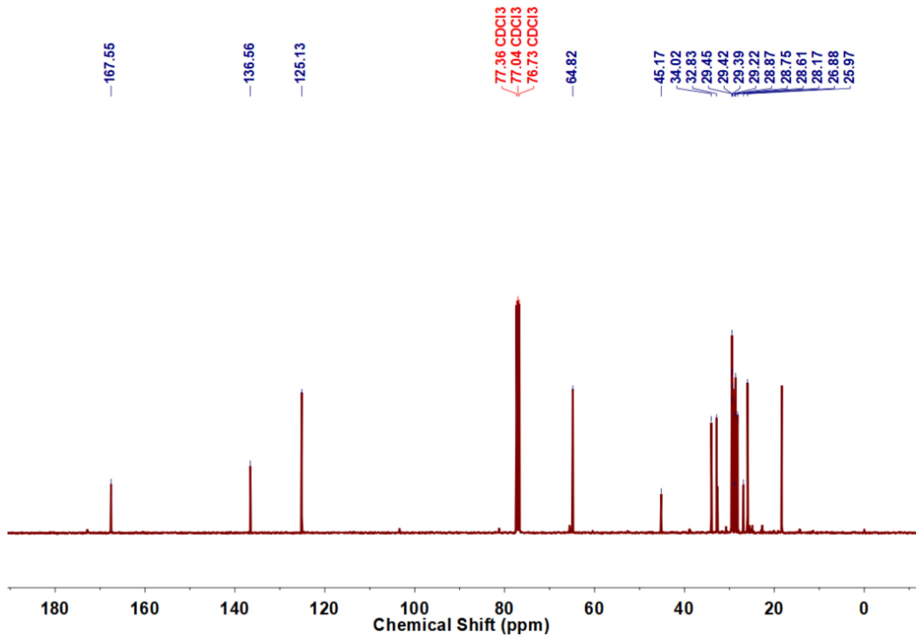

Supplementary Fig. 4 ^{13}C NMR spectrum ($CDCl_3$, room temperature, 400 MHz) of compound 3.

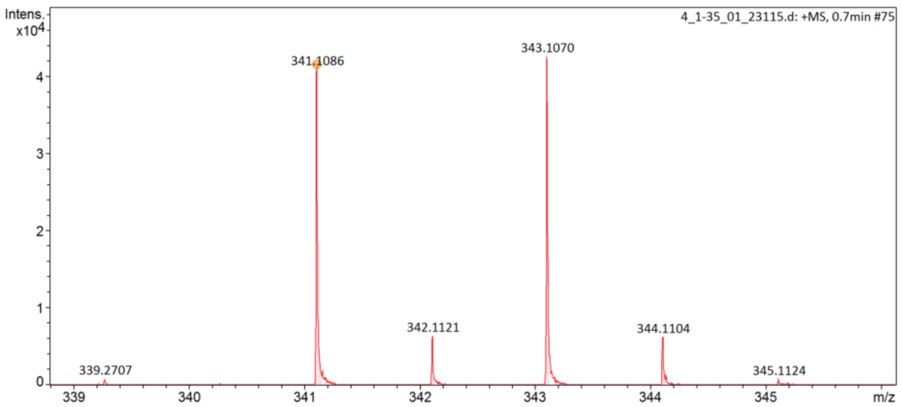

Supplementary Fig. 5 Electrospray ionization mass spectrum of compound 3.

Synthesis of 3,6,9,12,15,18-hexaoxaicosane-1,20-diyl bis(4-methylbenzenesulfonate) (4)

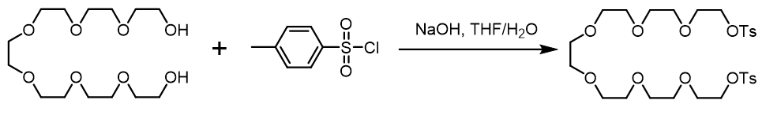

4

Compounds 4 was prepared according to the established methods[1]. Heptaethylene glycol (50.00 g, 153.24 mmol) and 4-toluenesulfonyl chloride (73.04 g, 383.11 mmol) were mixed in tetrahydrofuran (200 mL) in a flask. Then added sodium hydroxide aqueous solution (24.52 g, 612.96 mmol). After the addition, the reaction was stirred overnight at room temperature. The solvent was evaporated and the residue was dissolved in ethyl acetate and washed with water and brine. The mixture was concentrated under vacuum to afford compound 4 as a colorless oil (40.96 g, 84%). The ^{1}H NMR spectrum of compound 4 is shown in Supplementary Fig. 6. ^{1}H NMR ($CDCl_3$, room temperature, 400 MHz) δ (ppm): 7.77–7.75 (m, 4H), 7.33–7.31 (m, 4H), 4.14–4.11 (m, 4H), 3.66–3.64 (m, 4H), 3.63–3.59 (m, 12H), 3.55 (s, 8H),2.42 (s, 6H). The ^{13}C NMR spectrum of compound 4 is shown in Supplementary Fig. 7. ^{13}C NMR ($CDCl_3$, room temperature, 400 MHz) δ (ppm): 144.84, 132.95, 129.85, 127.96, 70.68, 70.54, 70.49, 70.48, 70.45, 69.29, 68.65, 21.64. HRESIMS is shown in Supplementary Fig. 8: *m*/*z* calcd for $C_{28}H_{42}O_{12}S_2$, 657.2010 [M + Na]$^+$; found 657.1997 [M + Na]$^+$.

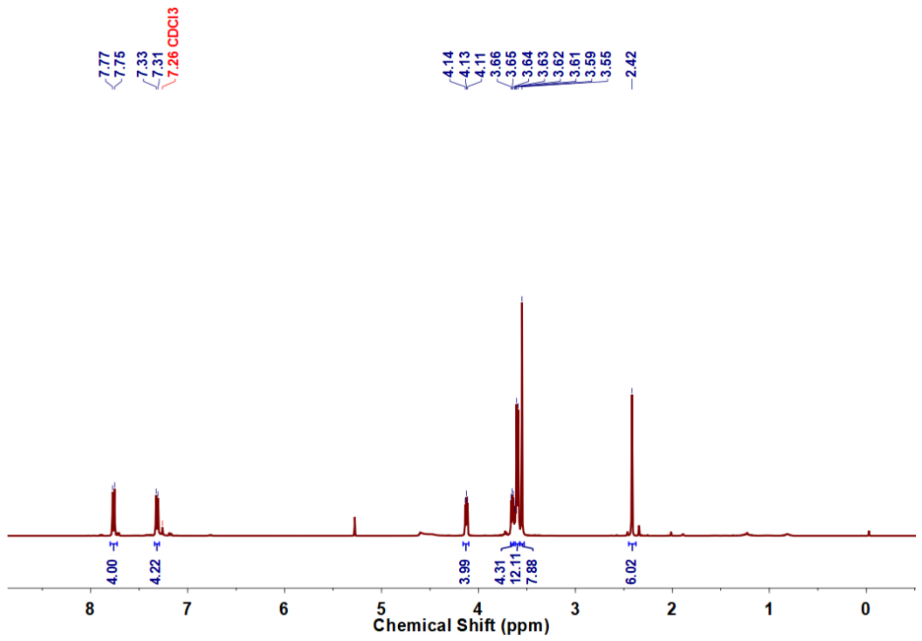

Supplementary Fig. 6 ^{1}H NMR spectrum ($CDCl_3$, room temperature, 400 MHz) of compound 4.

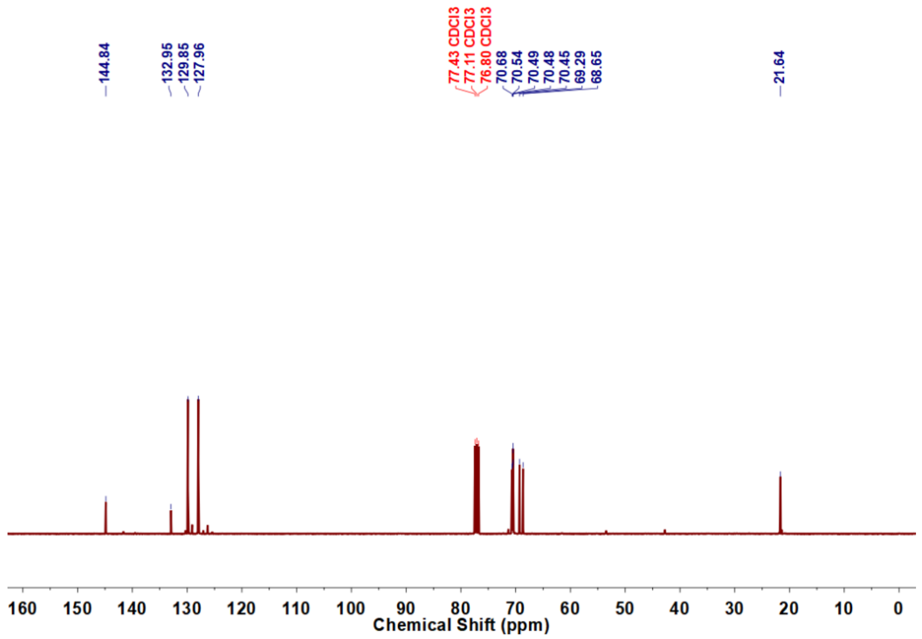

Supplementary Fig. 7 ^{13}C NMR spectrum ($CDCl_3$, room temperature, 400 MHz) of compound 4.

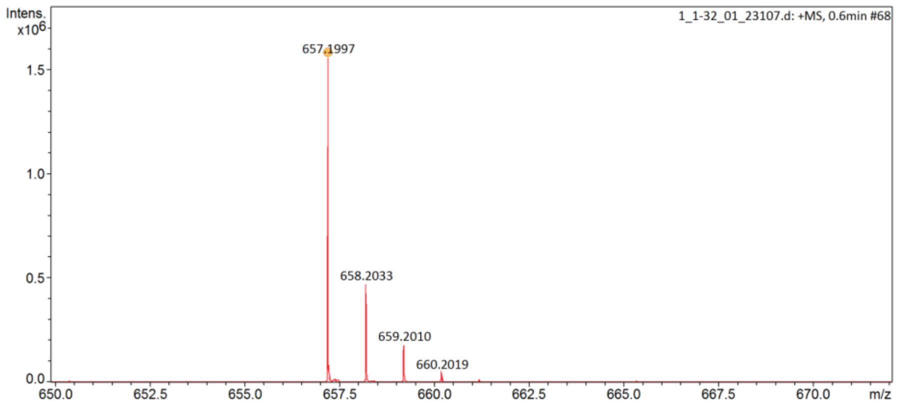

Supplementary Fig. 8 Electrospray ionization mass spectrum of compound 4.

Synthesis of methyl 2,3,5,6,8,9,11,12,14,15,17,18,20,21-tetradecahydrobenzo[b][1,4,7,10,13,16,19,22]octaoxacyclotetracosine-24-carboxylate (5)

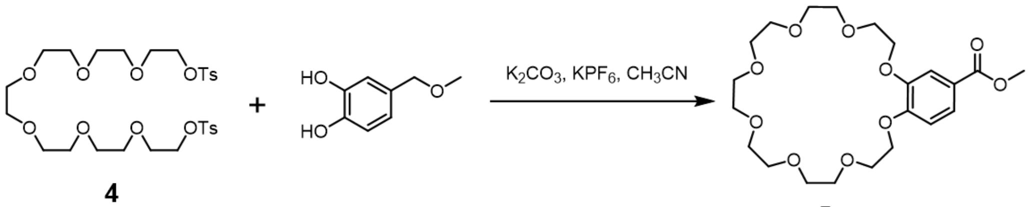

4

5

Compounds 5 was prepared according to the established methods[2]. Compound 4 (48.62 g, 76.60 mmol) and methyl 3,4-dihydroxybenzoate (12.88 g, 76.60 mmol) were mixed in acetonitrile (500 mL) in a flask. Then added K_2CO_3 (42.35 g, 306.40 mmol) and KPF_6 (21.15 g, 114.90 mmol), the reaction was heated to 85 °C with a magnetic stirring bar. The reaction was kept for 48 h, followed by cooled to ambient temperature and then the solvent was evaporated, the residue was dissolved in chloroform and washed with water and brine. The mixture was purified via gel chromatography (CH_3OH/CH_2Cl_2, 1:200 *v*/*v*) to afford compound 5 as a light-yellow oil (28.08 g, 80%). The ^{1}H NMR spectrum of compound 5 is shown in Supplementary Fig. 9. ^{1}H NMR ($CDCl_3$, room temperature, 400 MHz) δ (ppm): 7.66–7.63 (m, 1H), 7.54 (d, J = 2.0 Hz, 1H), 6.87 (d, J = 8.4 Hz, 1H), 4.21–4.18 (m, 4H), 3.93–3.90 (m, 4H), 3.87 (s, 3H), 3.79–3.77 (m, 4H), 3.71–3.64 (m, 16H). The ^{13}C NMR spectrum of compound 5 is shown in Supplementary Fig. 10. ^{13}C NMR ($CDCl_3$, room temperature, 400 MHz) δ (ppm): 166.81, 152.95, 148.29, 123.97, 122.95, 114.74, 112.42, 71.25, 71.17, 70.90, 70.88, 70.83, 70.72, 70.70, 69.69, 69.56, 69.35, 69.11, 51.98. HRESIMS is shown in Supplementary Fig. 11: *m*/*z* calcd for $C_{22}H_{34}O_{10}$, 481.2044 $[M + Na]^+$; found 481.2046 $[M + Na]^+$.

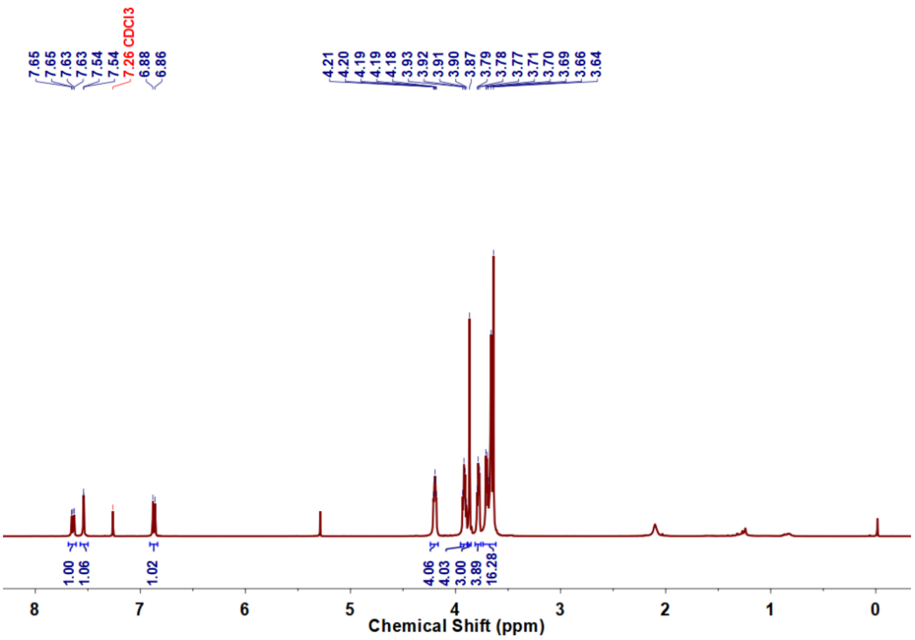

Supplementary Fig. 9 ^{1}H NMR spectrum ($CDCl_3$, room temperature, 400 MHz) of compound 5.

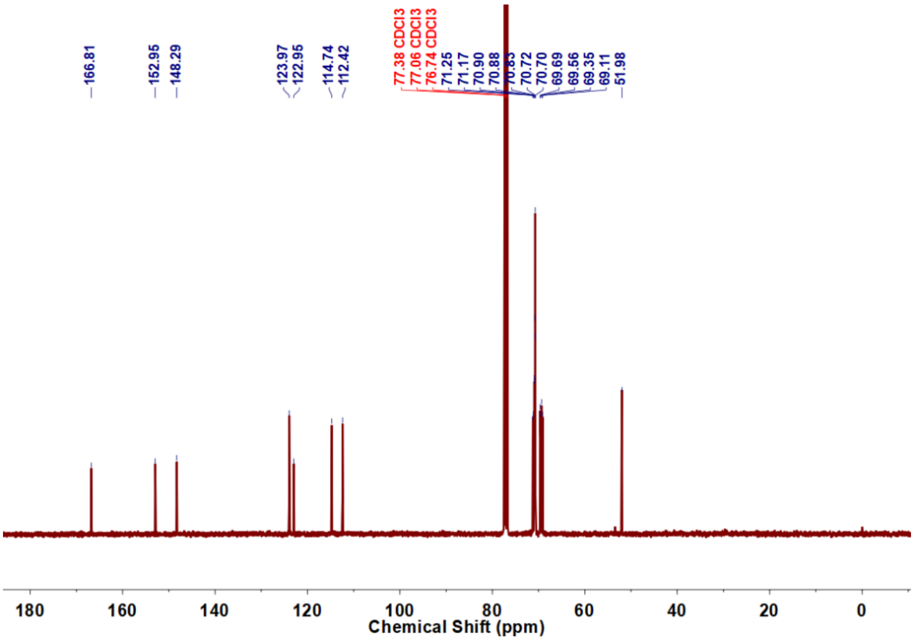

Supplementary Fig. 10 ^{13}C NMR spectrum ($CDCl_3$, room temperature, 400 MHz) of compound 5.

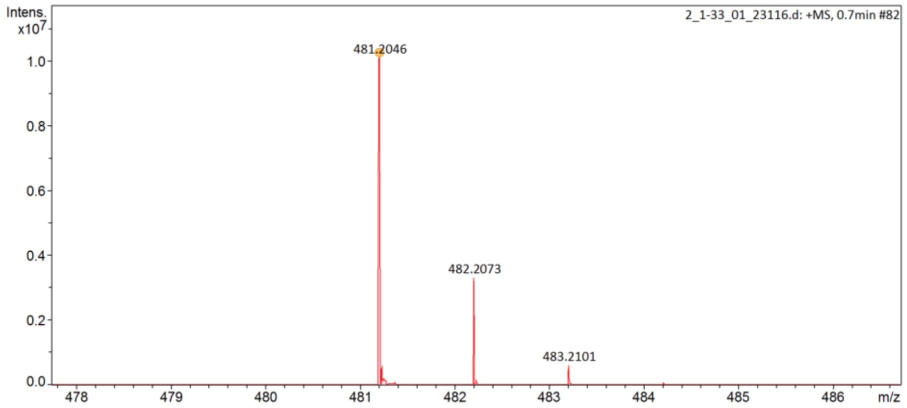

Supplementary Fig. 11 Electrospray ionization mass spectrum of compound 5.

Synthesis of 2,3,5,6,8,9,11,12,14,15,17,18,20,21-tetradecahydrobenzo[b][1,4,7,10,13,16,19,22]octaoxacyclotetracosine-24-carboxylic acid (6)

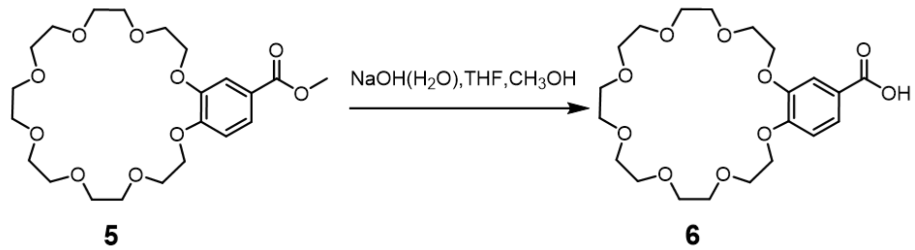

Compounds 6 were prepared according to the established methods[2]. Compound 5 (5.00 g, 10.90 mmol) and NaOH (1.74 g, 43.60 mmol) were mixed in tetrahydrofuran (30 mL) in a flask. Then added H_2O (30 mL) and CH_3OH (15 mL). After the addition, the reaction was stirred overnight at room temperature. The solvent was evaporated and the residue was dissolved in ethyl acetate and washed with water and brine. The mixture was concentrated under vacuum to afford compound 6 as a light-yellow oil (4.80 g, 99%). The ^{1}H NMR spectrum of compound 6 is shown in Supplementary Fig. 12. ^{1}H NMR ($CDCl_3$, room temperature, 400 MHz) δ (ppm): 7.74–7.71 (m, 1H), 7.59 (d, J = 2.0 Hz, 1H), 6.90 (d, J = 8.5 Hz, 1H), 4.24–4.20 (m, 4H), 3.96–3.92 (m, 4H), 3.82–3.78 (m, 4H), 3.73–3.66 (m, 16H). The ^{13}C NMR spectrum of compound 6 is shown in Supplementary Fig. 13. ^{13}C NMR ($CDCl_3$, room temperature, 400 MHz) δ (ppm): 170.46, 153.32, 148.25, 124.69, 122.54, 114.99, 112.31, 71.23, 71.14, 70.89, 70.86, 70.84, 70.83, 70.71, 69.72, 69.57, 69.26, 69.07. HRESIMS is shown in Supplementary Fig. 14: *m/z* calcd for $C_{21}H_{32}O_{10}$, 467.1888 $[M + Na]^+$; found 467.1873 $[M + Na]^+$.

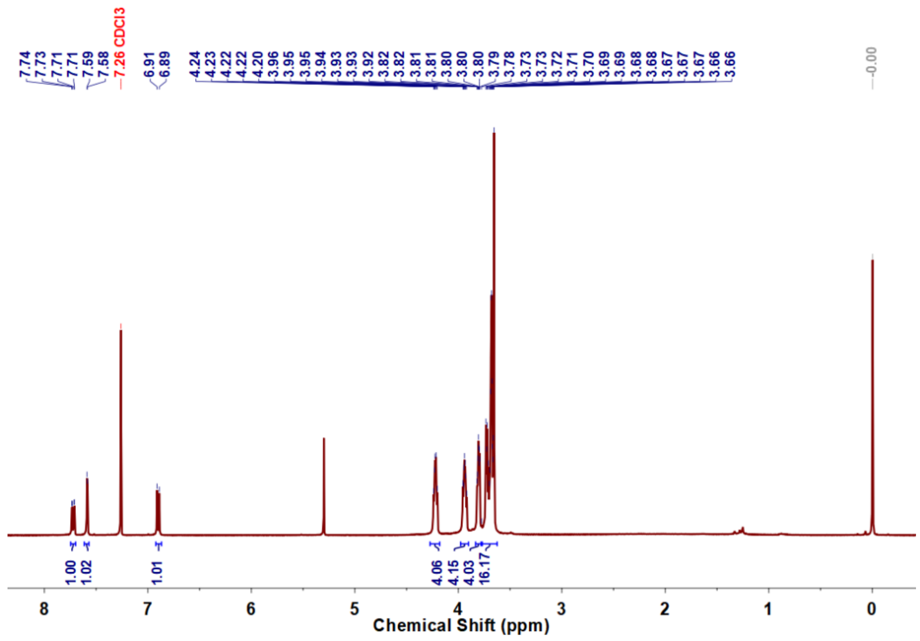

Supplementary Fig. 12 ^{1}H NMR spectrum ($CDCl_3$, room temperature, 400 MHz) of compound 6.

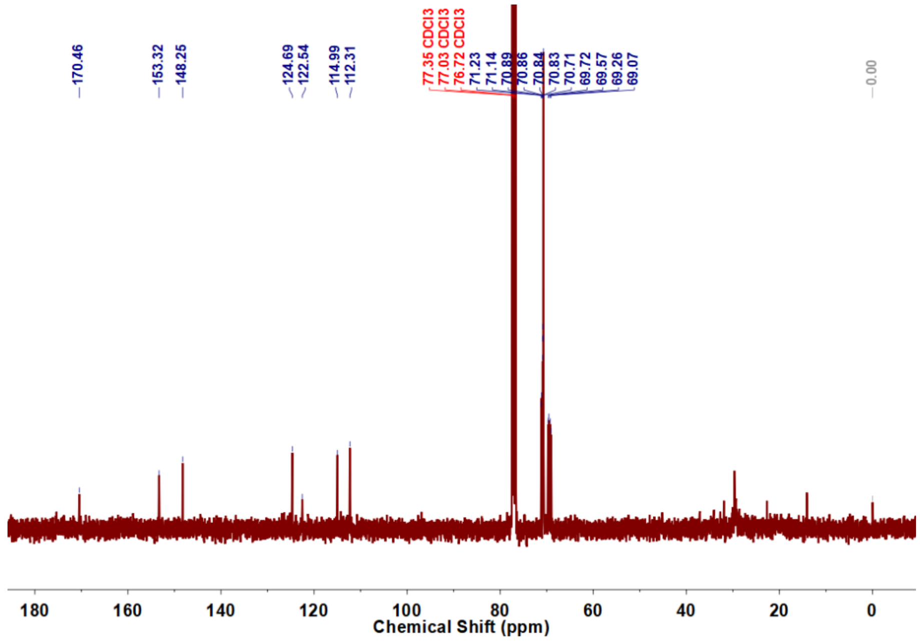

Supplementary Fig. 13 ^{13}C NMR spectrum ($CDCl_3$, room temperature, 400 MHz) of compound 6.

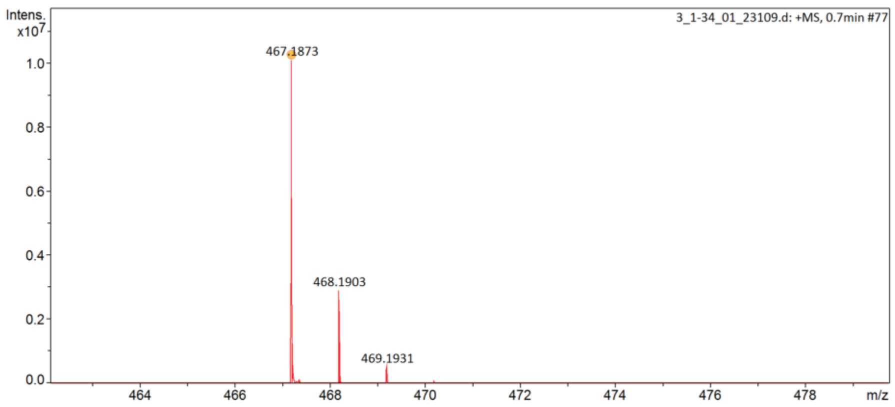

Supplementary Fig. 14 Electrospray ionization mass spectrum of compound 6.

Synthesis of 11-(methacryloyloxy)undecyl 2,3,5,6,8,9,11,12,14,15,17,18,20,21-tetradecahydrobenzo[b][1,4,7,10,13,16,19,22]octaoxacyclotetracosine-24-carboxylate (1)

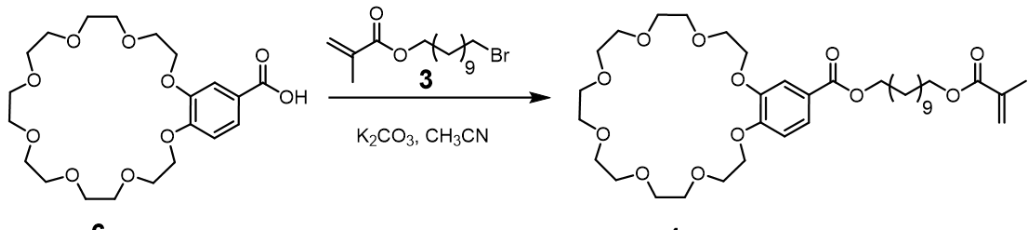

A stirred solution of compound 6 (1.00 g, 2.25 mmol), compound 3 (1.08 g, 3.38 mmol) and K_2CO_3 (0.62 g, 4.50 mmol) in acetonitrile (10 mL), in a round-bottomed flask with a magnetic stir-bar was heated to 60 °C via an oil bath. The reaction was kept for 48 h and then cooled to ambient temperature. The mixture was filtered and the filtrate was evaporated. The crude product was further purified by gel chromatography (CH_3OH/CH_2Cl_2, 1:300 *v*/*v*) to afford compound 1 as a light-yellow oil (0.80 g, 52%). The ^{1}H NMR spectrum of compound 1 is shown in Supplementary Fig. 15. ^{1}H NMR ($CDCl_3$, room temperature, 400 MHz) δ (ppm): 7.62–7.66 (m, 1H), 7.55 (d, J = 2.0 Hz, 1H), 6.87 (d, J = 8.5 Hz, 1H), 6.08–6.09 (m, 1H), 5.52–5.54 (m, 1H), 4.26 (t, J = 6.7 Hz, 2H), 4.18–4.21 (m, 4H), 4.12 (t, J = 6.7 Hz, 2H), 3.95–3.86 (m, 4H), 3.82–3.74 (m, 4H), 3.74–3.68 (m, 4H), 3.68–3.60 (m, 12H), 1.93 (t, J = 1.3 Hz, 3H), 1.78–1.61 (m, 4H), 1.45–1.23 (m, 14H). The ^{13}C NMR spectrum of compound 1 is shown in Supplementary Fig. 16. ^{13}C NMR ($CDCl_3$, room temperature, 400 MHz) δ (ppm): 167.56, 166.40, 166.37, 152.94, 152.86, 148.31, 148.27, 136.56, 125.13, 123.89, 123.35, 114.91, 112.43, 71.32, 71.23, 70.97, 70.95, 70.92, 70.89, 70.87, 70.78, 70.76, 70.68, 70.62,

69.75, 69.66, 69.60, 69.53, 69.44, 69.17, 69.00, 68.72, 64.97, 64.82, 53.44, 29.50, 29.48, 29.29, 29.23, 28.77, 28.60, 26.04, 25.98, 18.34. HRESIMS is shown in Supplementary Fig. 17: *m*/*z* calcd for $C_{36}H_{58}O_{12}$, 705.3826 $[M + Na]^+$; found 705.3820 $[M + Na]^+$.

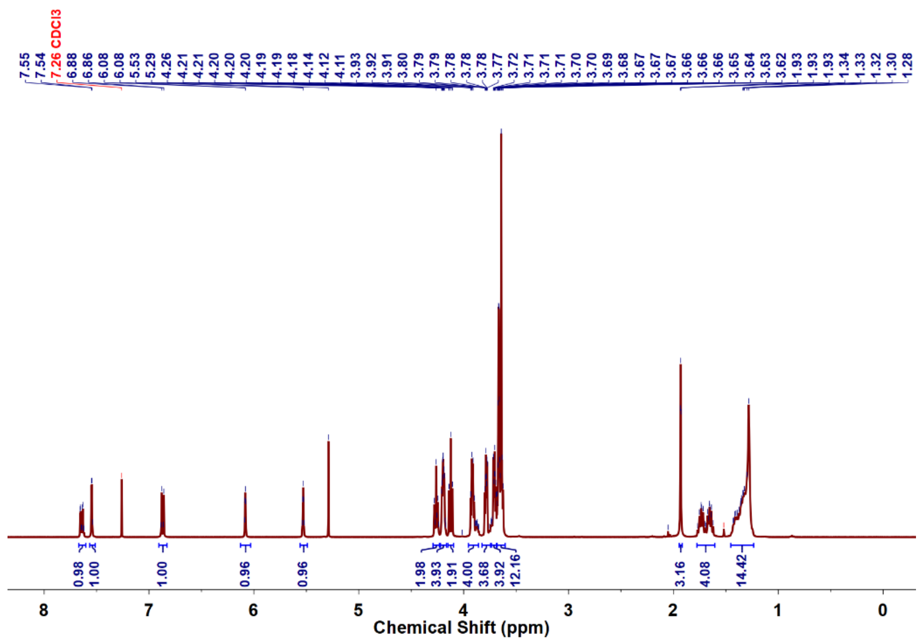

Supplementary Fig. 15 ^1H NMR spectrum ($CDCl_3$, room temperature, 400 MHz) of compound 1.

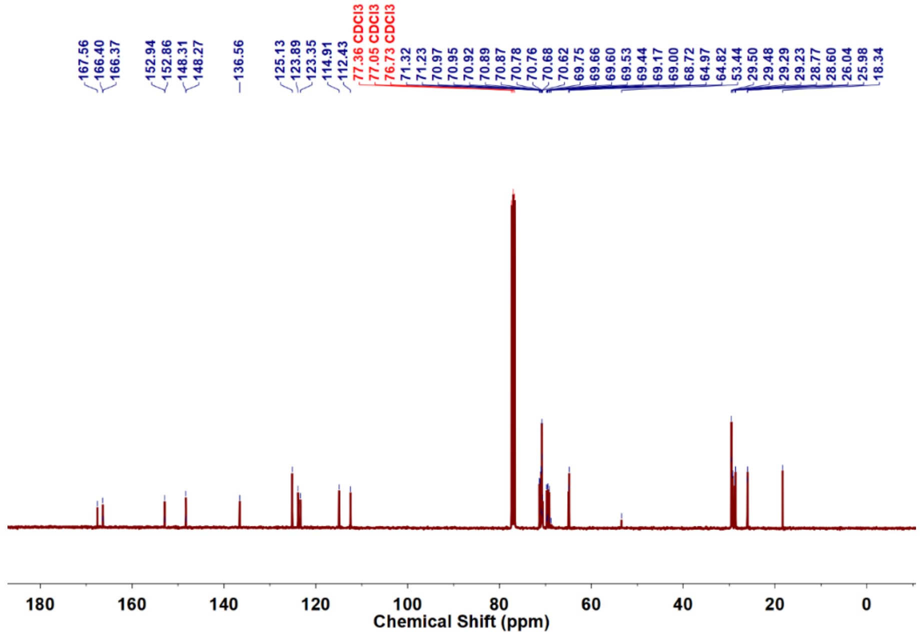

Supplementary Fig. 16 ^{13}C NMR spectrum ($CDCl_3$, room temperature, 400 MHz) of compound 1.

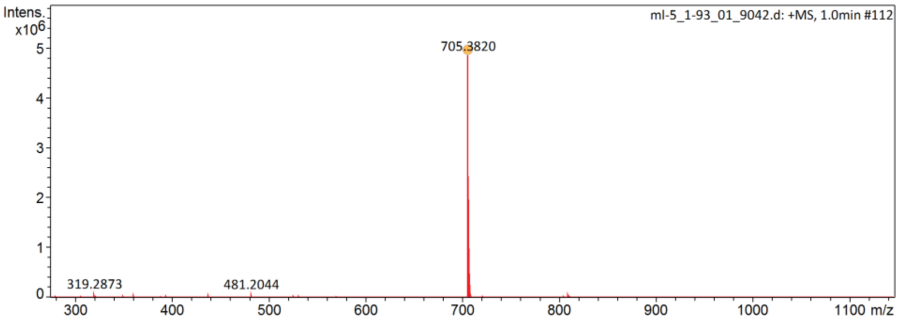

Supplementary Fig. 17 Electrospray ionization mass spectrum of compound 1.

Synthesis of 10-aminodecan-1-ol (7)

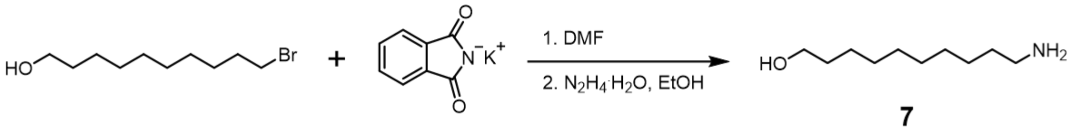

1) Within a round-bottomed flask, a solution of phthalimide (18.70 g, 101.20 mmol) and 10-bromo-1-undecanol (20.00 g, 84.30 mmol) in DMF (280 mL) was heated to 85 °C with a magnetic stirring bar. The reaction was kept for 12 h, followed by cooled to ambient temperature and then the mixture was filtered. 2) The solvent of the filtrate was removed under vacuum and the residue was dissolved in EtOH (200 mL). Then hydrazine hydrate (80%, 15.83 g) was added and the mixture was heated to 85 °C under stirring until the white precipitate formed. The EtOH in the mixture was removed and the white precipitate was dissolved by 6.0 M HCl. The aqueous solution was alkalized with NaOH and extracted with CH_2Cl_2. Finally, the organic phase was dried using Na_2SO_4 and the solvent was removed under vacuum to afford compound 7 as a white powder (12.60 g, 86%). Mp: 66.9–67.6 °C. The ^{1}H NMR spectrum of compound 7 is shown in Supplementary Fig. 18. ^{1}H NMR ($CDCl_3$, room temperature, 400 MHz) δ (ppm): 3.66–3.57 (m, 2H), 2.66 (t, J = 7.0 Hz, 2H), 1.53–1.59 (m, 6H), 1.42 (t, J = 7.0 Hz, 2H), 1.39–1.22 (m, 13H). The ^{13}C NMR spectrum of compound 7 is shown in Supplementary Fig. 19. ^{13}C NMR ($CDCl_3$, room temperature, 400 MHz) δ (ppm): 77.35, 77.03, 76.71, 62.94, 42.24, 33.80, 32.82, 29.53, 29.47, 29.41, 26.87, 25.75. HRESIMS is shown in Supplementary Fig. 20: m/z calcd for $C_{10}H_{23}NO$, 174.1852 $[M + H]^+$; found 174.1852 $[M + H]^+$.

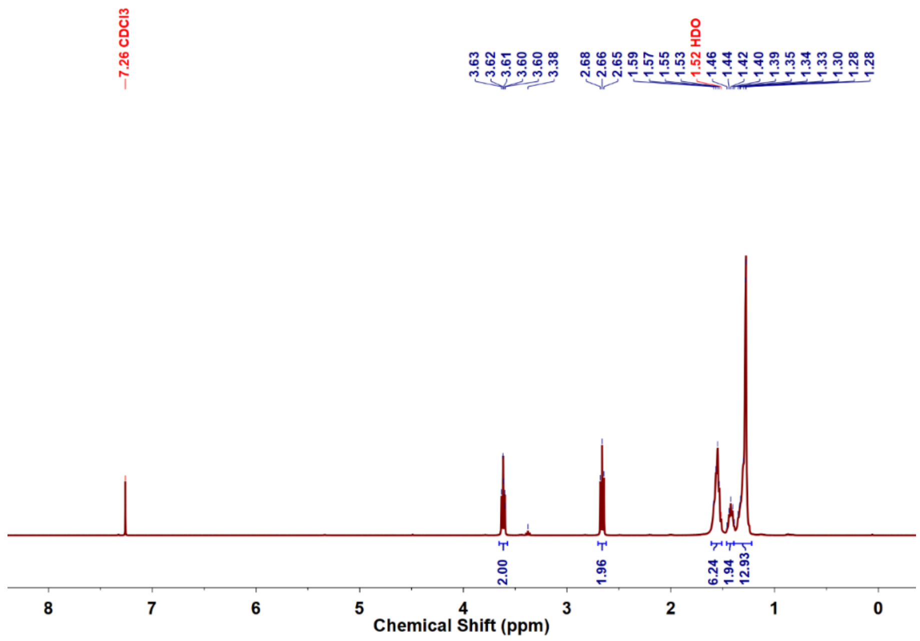

Supplementary Fig. 18 ^{1}H NMR spectrum ($CDCl_3$, room temperature, 400 MHz) of compound 7.

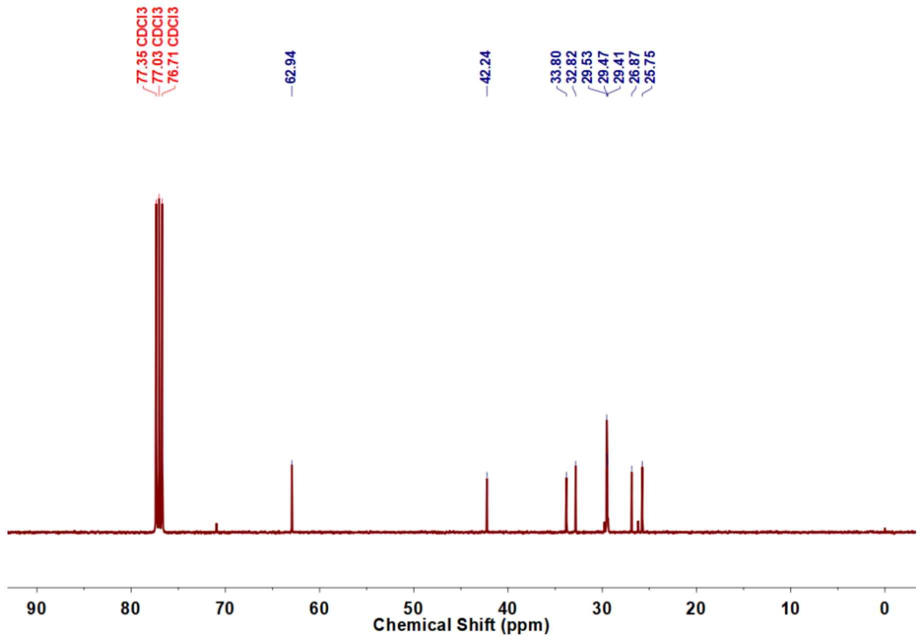

Supplementary Fig. 19 ^{13}C NMR spectrum ($CDCl_3$, room temperature, 400 MHz) of compound 7.

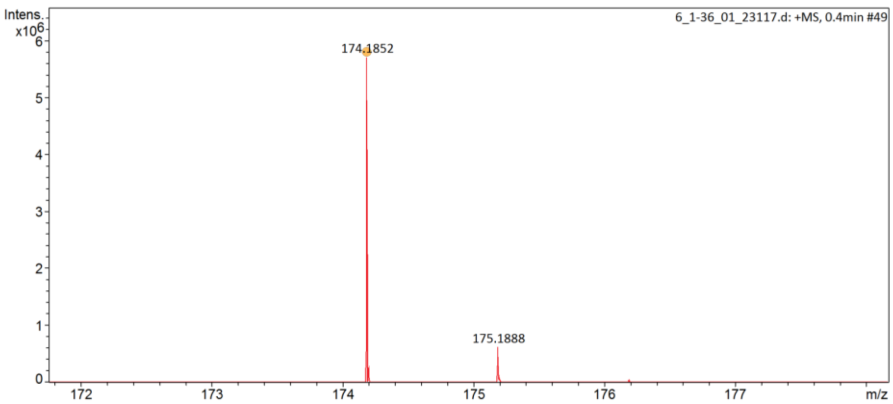

Supplementary Fig. 20 Electrospray ionization mass spectrum of compound 7.

Synthesis of tert-butyl (4-hydroxy-3,5-dimethylbenzyl)(10-hydroxydecyl)carbamate (8)

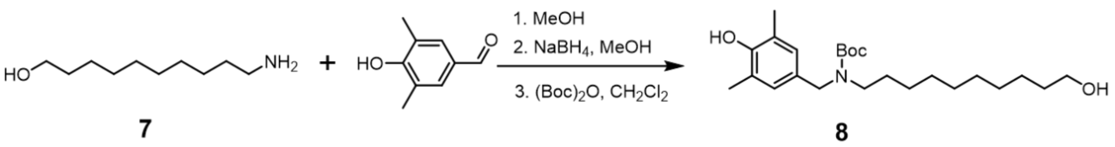

1) A solution of compound 7 (10.00 g, 57.74 mmol) and 4-hydroxy-3,5-dimethylbenzaldehyde (8.67 g, 57.74 mmol) in MeOH (200 mL), in a round-bottomed flask with a magnetic stir-bar was heated to 75 °C via an oil bath under N_2. The reaction was kept for 12 h and then cooled down to room temperature. 2) $NaBH_4$ (4.37 g, 115.48 mmol) was slowly added and the mixture was stirred for another 24 h at room temperature. 3) The solvent was evaporated and the residue was dissolved in CH_2Cl_2 and washed with water and brine. The organic phase was collected. The di-*tert*-butyl dicarbonate (15.12 g, 69.29 mmol) was then added into the collected mixture which was kept stirring for 12 h. Finally, the solvent was removed under vacuum and the mixture was purified via gel chromatography (petroleum ether/CH_2Cl_2, 3:1 *v*/*v*) to afford compound 8 as a reddish-brown oil (14.40 g, 61%). The ^{1}H NMR spectrum of compound 8 is shown in Supplementary Fig. 21. ^{1}H NMR ($CDCl_3$, room temperature, 400 MHz) δ (ppm): 6.83 (s, 2H), 4.83 (s, 1H), 4.30 (d, J = 12.3 Hz, 2H), 3.63 (t, J = 6.6 Hz, 2H), 3.11 (d, J = 24.3 Hz, 2H), 2.22 (s, 6H), 1.73–1.40 (m, 16H), 1.21–1.35 (m, 13H). The ^{13}C NMR spectrum of compound 8 is shown in Supplementary Fig. 22. ^{13}C NMR ($CDCl_3$, room temperature, 400 MHz) δ (ppm): 156.13, 151.42, 129.86, 128.07, 127.58, 123.22, 79.42, 63.02, 49.63, 49.12, 46.09, 32.74, 29.49, 29.44, 29.37, 29.29, 28.51, 26.81, 25.71, 16.06. HRESIMS is

shown in Supplementary Fig. 23: *m/z* calcd for $C_{24}H_{41}NO_4$, 430.2934 [M + Na]$^+$; found 430.2925 [M + Na]$^+$.

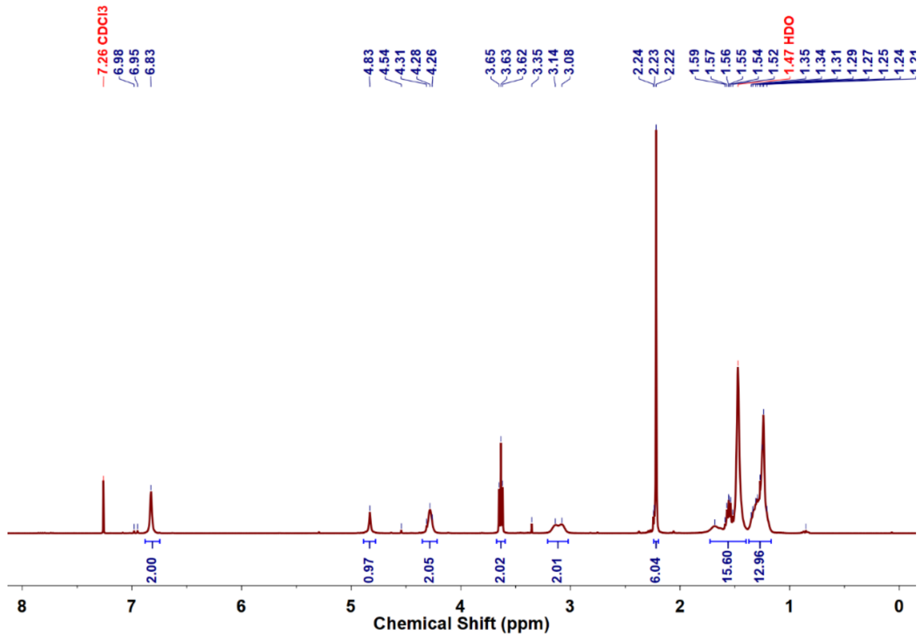

Supplementary Fig. 21 ^{1}H NMR spectrum ($CDCl_3$, room temperature, 400 MHz) of compound 8.

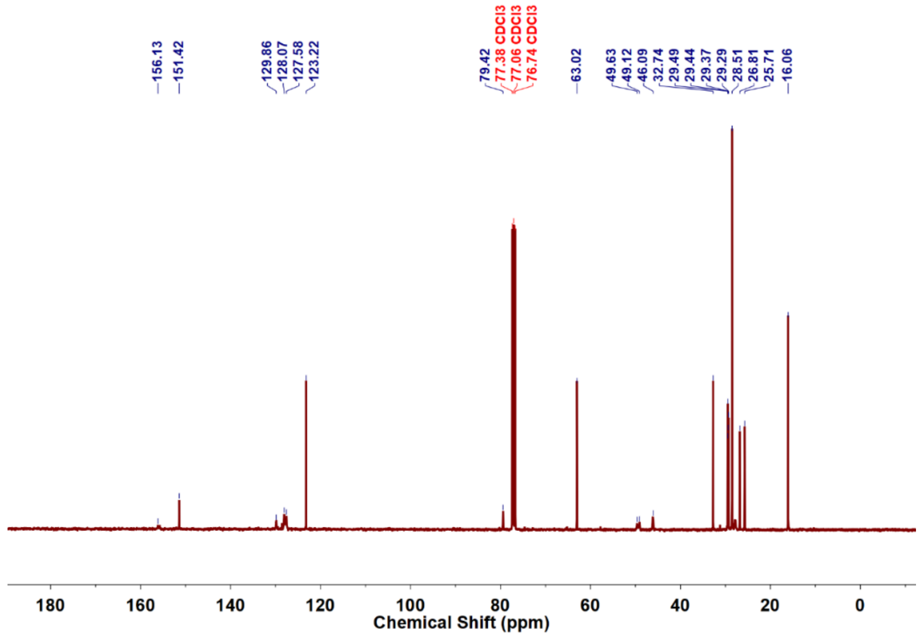

Supplementary Fig. 22 ^{13}C NMR spectrum ($CDCl_3$, room temperature, 400 MHz) of compound 8.

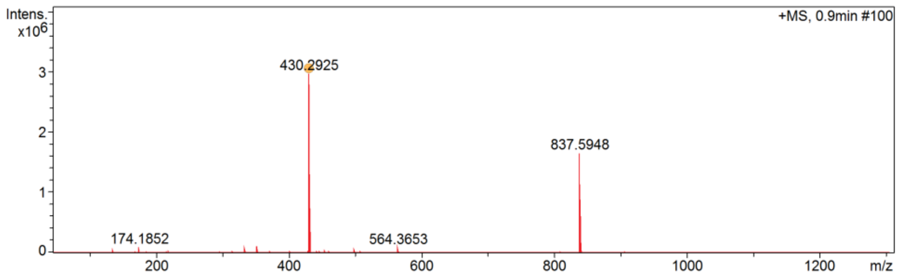

Supplementary Fig. 23 Electrospray ionization mass spectrum of compound 8.

Synthesis of 11-(4-(((tert-butoxycarbonyl)(10-hydroxydecyl)amino)methyl)-2,6-dimethylphenoxy)undecyl methacrylate (9)

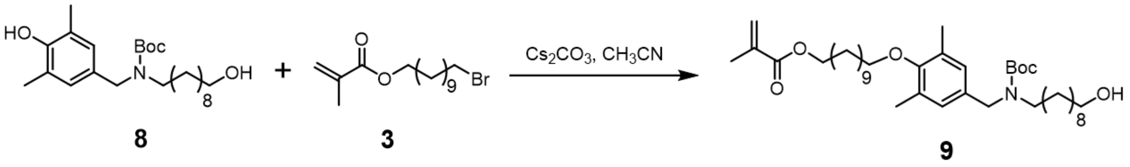

A stirred solution of compound 8 (1.00 g, 2.45 mmol), compound 3 (1.17 g, 3.68 mmol) and Cs_2CO_3 (1.60 g, 4.90 mmol) in acetonitrile (10 mL), in a round-bottomed flask with a magnetic stir-bar was heated to reflux for 72 h under N_2 and then cooled to room temperature. The mixture was filtered and the filtrate was evaporated. The crude product was purified by gel chromatography (petroleum ether/ethyl acetate, 10:1 *v*/*v*) to afford compound 9 as a light-yellow oil (0.96 g, 61%). The ^1H NMR spectrum of compound 9 is shown in Supplementary Fig. 24. ^1H NMR ($CDCl_3$, room temperature, 400 MHz) δ (ppm): 6.84 (s, 2H), 6.09–6.10 (m, 1H), 5.53–5.55 (m, 1H), 4.29 (s, 2H), 4.09–4.15 (m, 2H), 3.72 (t, J = 6.6 Hz, 2H), 3.63 (t, J = 6.6 Hz, 2H), 3.12 (d, J = 24.8 Hz, 2H), 2.24 (s, 6H), 1.94 (t, J = 1.3 Hz, 3H), 1.75–1.82 (m, 2H), 1.63–1.70 (m, 2H), 1.61–1.51 (m, 4H), 1.50–1.16 (m, 38H). The ^{13}C NMR spectrum of compound 9 is shown in Supplementary Fig. 25. ^{13}C NMR ($CDCl_3$, room temperature, 400 MHz) δ (ppm): 167.57, 155.09, 136.56, 133.61, 130.79, 128.03, 127.59, 125.14, 79.37, 72.35, 64.84, 63.00, 60.39, 49.15, 46.37, 32.79, 32.79, 30.43, 29.58, 29.55, 29.51, 29.49, 29.48, 29.38, 29.32, 29.24, 28.61, 28.48, 26.83, 26.16, 25.98, 25.72, 21.04, 18.33, 16.34, 14.20. HRESIMS is shown in Supplementary Fig. 26: *m*/*z* calcd for $C_{39}H_{67}NO_6$, 646.4968 $[M + H]^+$; found 646.5033 $[M + H]^+$; 668.4866 $[M + Na]^+$; found 668.4852 $[M + Na]^+$.

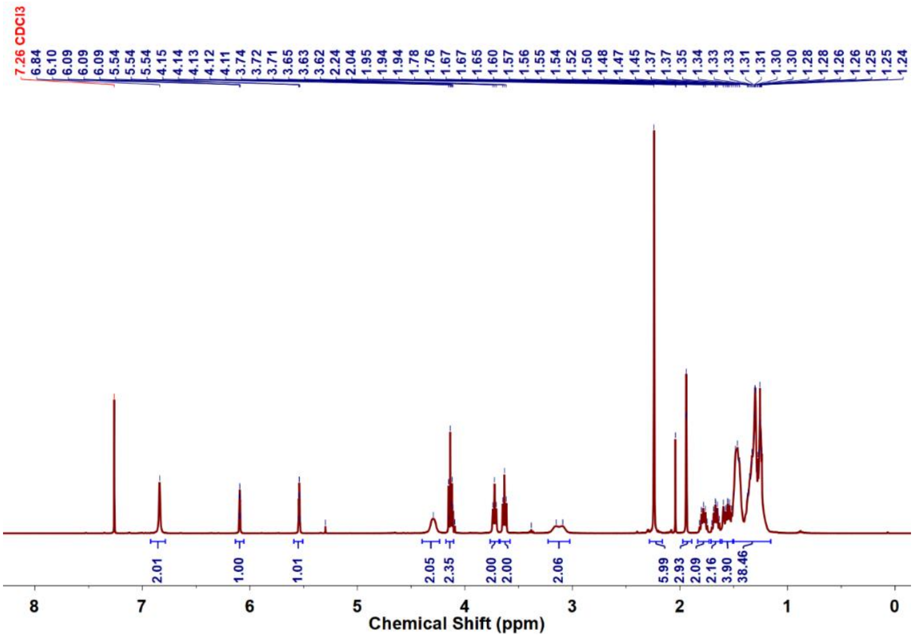

Supplementary Fig. 24 ^{1}H NMR spectrum ($CDCl_3$, room temperature, 400 MHz) of compound 9.

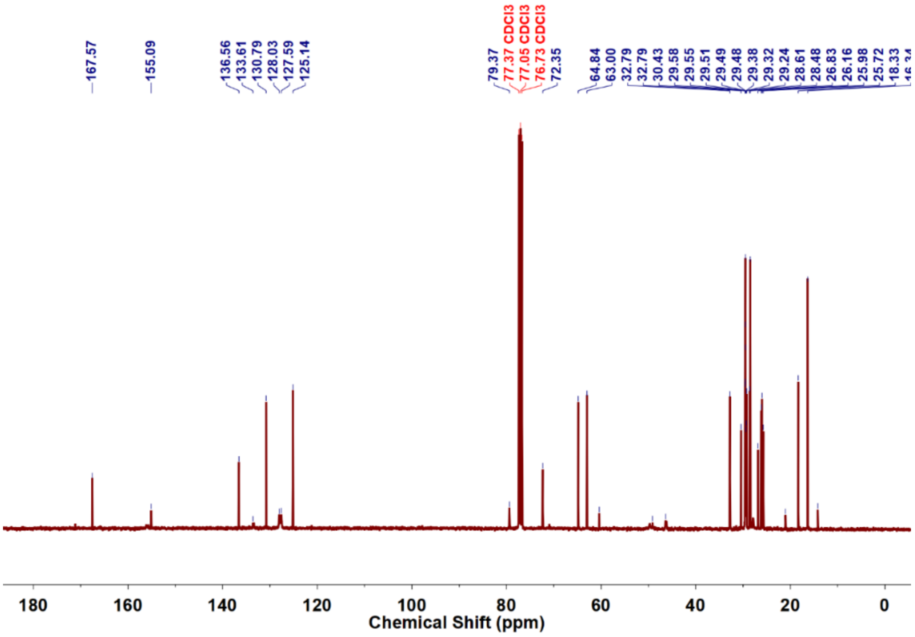

Supplementary Fig. 25 ^{13}C NMR spectrum ($CDCl_3$, room temperature, 400 MHz) of compound 9.

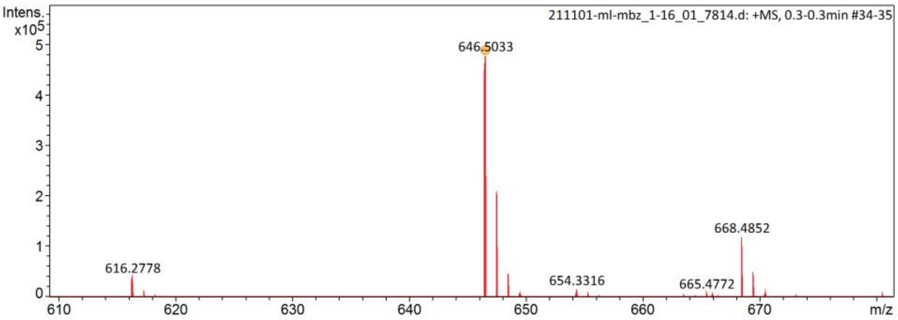

Supplementary Fig. 26 Electrospray ionization mass spectrum of compound 9.

Synthesis of 10-hydroxy-N-(4-((11-(methacryloyloxy)undecyl)oxy)-3,5-dimethylbenzyl)decan-1-aminium hexafluorophosphate (2)

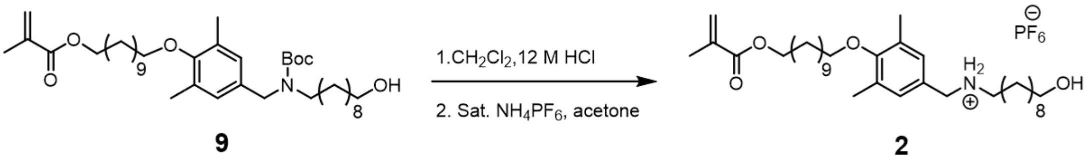

1) Compound 9 (1.00 g, 1.50 mmol) was dissolved in CH_2Cl_2 (15 mL) and 1.0 mL of con. HCl was subsequently dropwise added into the solution. The mixture was kept stirring for 12 h and then washed with water for three times. 2) The solvent was removed under vacuum and the residue was dissolved in 20 mL of acetone. Then 3 mL of sat. NH_4PF_6 aqueous solution was added and the mixture was stirred for another 12 h. Finally, the volatile solvent was evaporated and the residue suspended in water was extracted with CH_2Cl_2. The organic phase was dried using Na_2SO_4 and the solvent was removed under vacuum to afford compound 2 as a yellow waxy solid (0.87 g, 84%). The ^{1}H NMR spectrum of compound 2 is shown in Supplementary Fig. 27. ^{1}H NMR ($CDCl_3$, room temperature, 400 MHz) δ (ppm): 7.06 (s, 2H), 6.09 (t, J = 1.4 Hz, 1H), 5.53–5.55 (m, 1H), 4.11 (dd, J = 12.4, 5.7 Hz, 4H), 3.72 (t, J = 6.6 Hz, 2H), 3.57 (t, J = 6.6 Hz, 2H), 2.98–2.90 (m, 2H), 2.26 (s, 6H), 1.93 (t, J = 1.3 Hz, 3H), 1.83–1.62 (m, 6H), 1.43–1.51 (m, 4H), 1.41–1.21 (m, 25H). The ^{13}C NMR spectrum of compound 2 is shown in Supplementary Fig. 28. ^{13}C NMR ($CDCl_3$, room temperature, 400 MHz) δ (ppm): 167.69, 157.53, 136.52, 132.44, 130.39, 125.26, 124.41, 72.39, 64.90, 62.99, 51.93, 47.37, 32.44, 30.95, 30.37, 29.54, 29.49, 29.23, 29.00, 28.73, 28.62, 28.60, 26.08, 25.97, 25.94, 25.84, 25.41, 18.32, 16.22. HRESIMS is shown in Supplementary Fig. 29: m/z calcd for $C_{34}H_{60}NO_4$, 546.4517 $[M - PF_6]^+$; found 546.4522 $[M - PF_6]^+$.

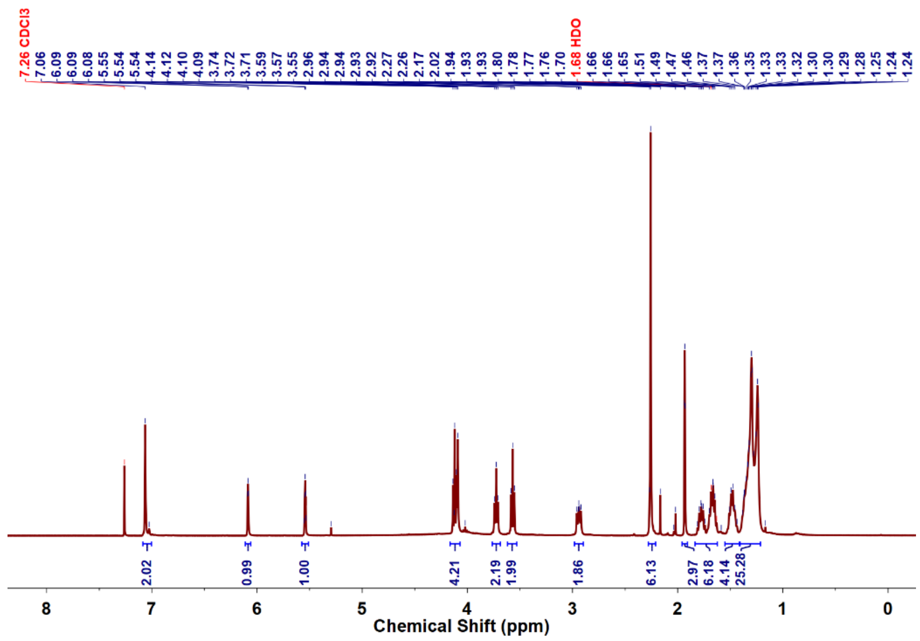

Supplementary Fig. 27 ^{1}H NMR spectrum ($CDCl_3$, room temperature, 400 MHz) of compound 2.

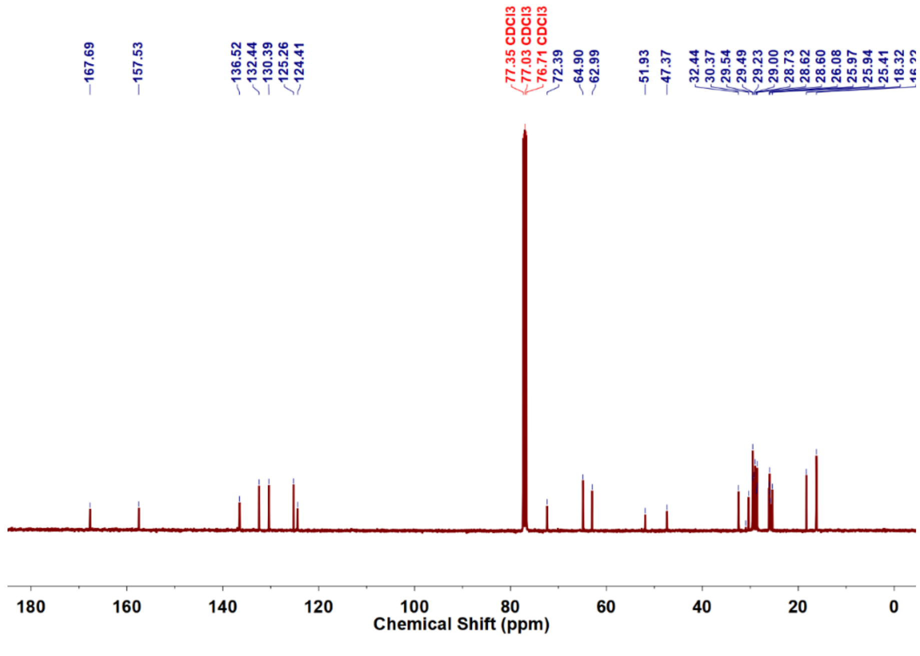

Supplementary Fig. 28 ^{13}C NMR spectrum ($CDCl_3$, room temperature, 400 MHz) of compound 2.

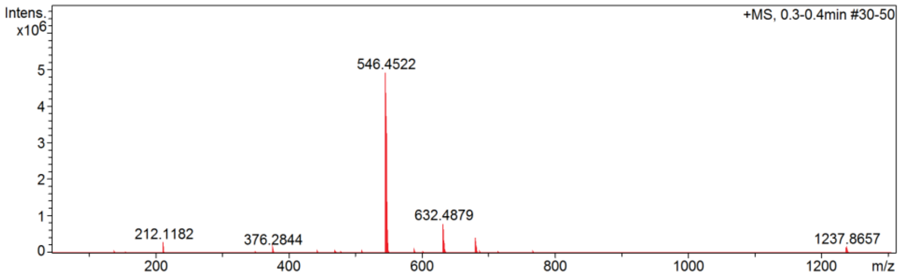

Supplementary Fig. 29 Electrospray ionization mass spectrum of compound 2.

Synthesis of [2]rotaxane

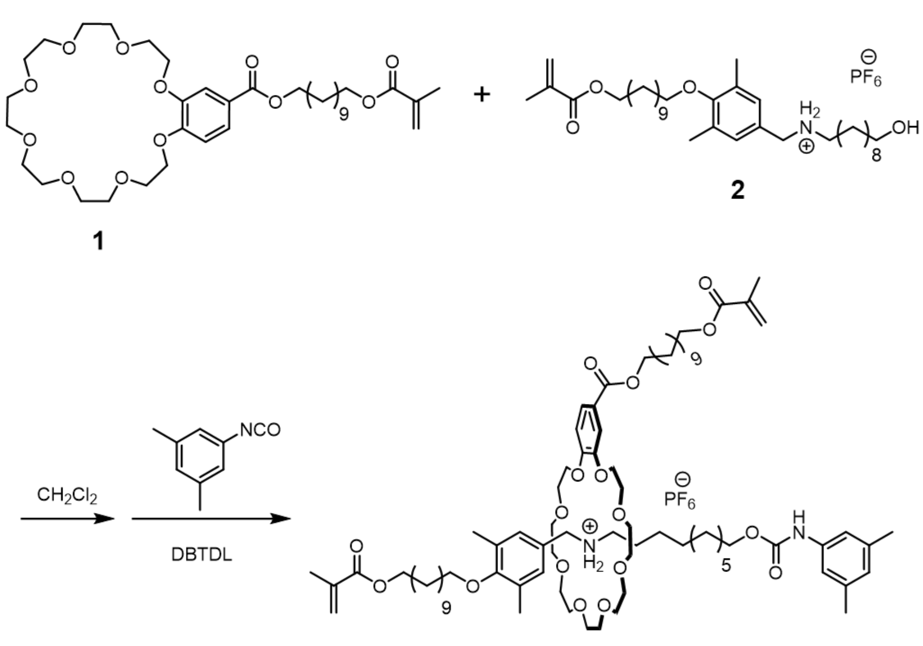

A stirred solution of compounds 1 (0.54 g, 0.79 mmol) and 2 (0.50 g, 0.72 mmol) in dry CH_2Cl_2 (30 mL) in a round-bottomed flask with a magnetic stir-bar was heated to reflux for 36 h under N_2. Upon cooling to room temperature, 3,5-dimethylphenyl isocyanate (0.21 g, 1.44 mmol) and 0.05 mL of dibutyltin dilaurate were added to the solution which was stirred at room temperature for another 12 h. The solution was evaporated and the residue was purified by gel chromatography (CH_2Cl_2/acetone, 300:1 *v*/*v*) to afford [2]rotaxane as a reddish-brown oil (0.96 g, 61%). The ^{1}H NMR spectrum of [2]rotaxane is shown in Supplementary Fig. 30. ^{1}H NMR ($CDCl_3$, room temperature, 400 MHz) δ (ppm): 7.64 (dd, J = 8.4, 2.0 Hz, 1H), 7.47 (d, J = 2.0 Hz, 1H), 7.01 (d, J = 17.1 Hz, 6H), 6.87 (d, J = 8.5 Hz, 1H), 6.76 (s, 1H), 6.68 (s, 1H), 6.13–6.05 (m, 2H), 5.53–5.54 (m, 2H), 4.40–4.06 (m, 15H), 3.94–3.83 (m, 4H), 3.70–3.32 (m, 23H), 3.10–3.18 (m, 2H), 2.27 (s,

6H), 2.19 (s, 6H), 1.93–1.94 (m, 6H), 1.82–1.23 (m, 53H). The ^{13}C NMR spectrum of [2]rotaxane is shown in Supplementary Fig. 31. ^{13}C NMR ($CDCl_3$, room temperature, 400 MHz) δ (ppm): 167.59, 166.22, 156.60, 153.85, 151.95, 147.57, 138.68, 137.97, 136.54, 131.19, 130.45, 127.54, 125.18, 124.97, 123.99, 123.38, 113.32, 111.67, 72.48, 71.14, 70.73, 70.67, 70.57, 70.46, 70.30, 68.38, 68.29, 65.18, 64.83, 51.76, 48.78, 30.45, 29.60, 29.54, 29.52, 29.50, 29.31, 29.24, 29.16, 29.10, 28.93, 28.77, 28.61, 26.59, 26.55, 26.18, 26.02, 25.98, 25.81, 21.39, 18.34, 16.31. HRESIMS is shown in Supplementary Fig. 32: *m*/*z* calcd for $C_{79}H_{127}NO_{17}PF_6$, 1375.9129 $[M - PF_6]^+$; found 1375.9139 $[M - PF_6]^+$.

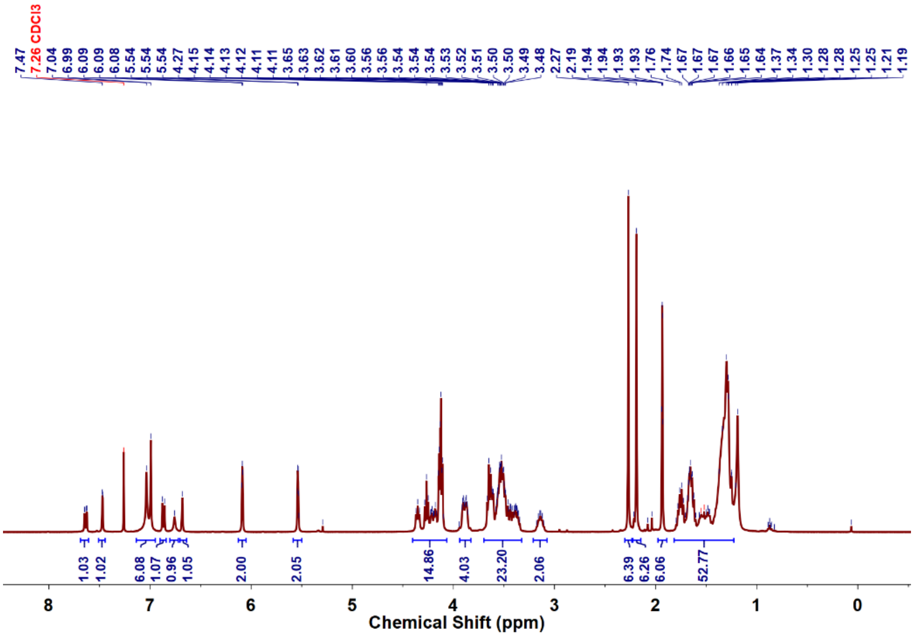

Supplementary Fig. 30 ^{1}H NMR spectrum ($CDCl_3$, room temperature, 400 MHz) of [2]rotaxane.

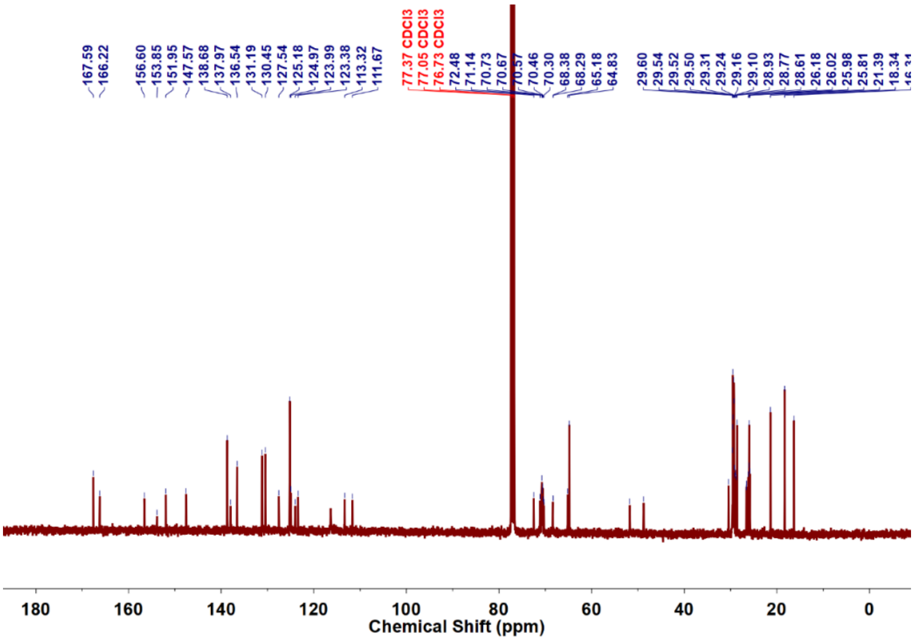

Supplementary Fig. 31 ^{13}C NMR spectrum ($CDCl_3$, room temperature, 400 MHz) of [2]rotaxane.

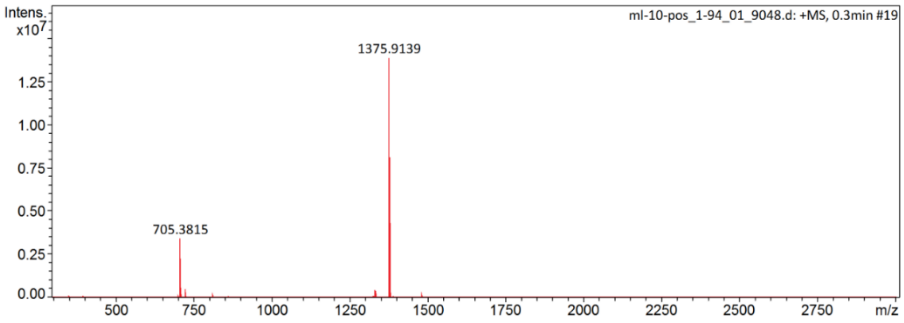

Supplementary Fig. 32 Electrospray ionization mass spectrum of [2]rotaxane.

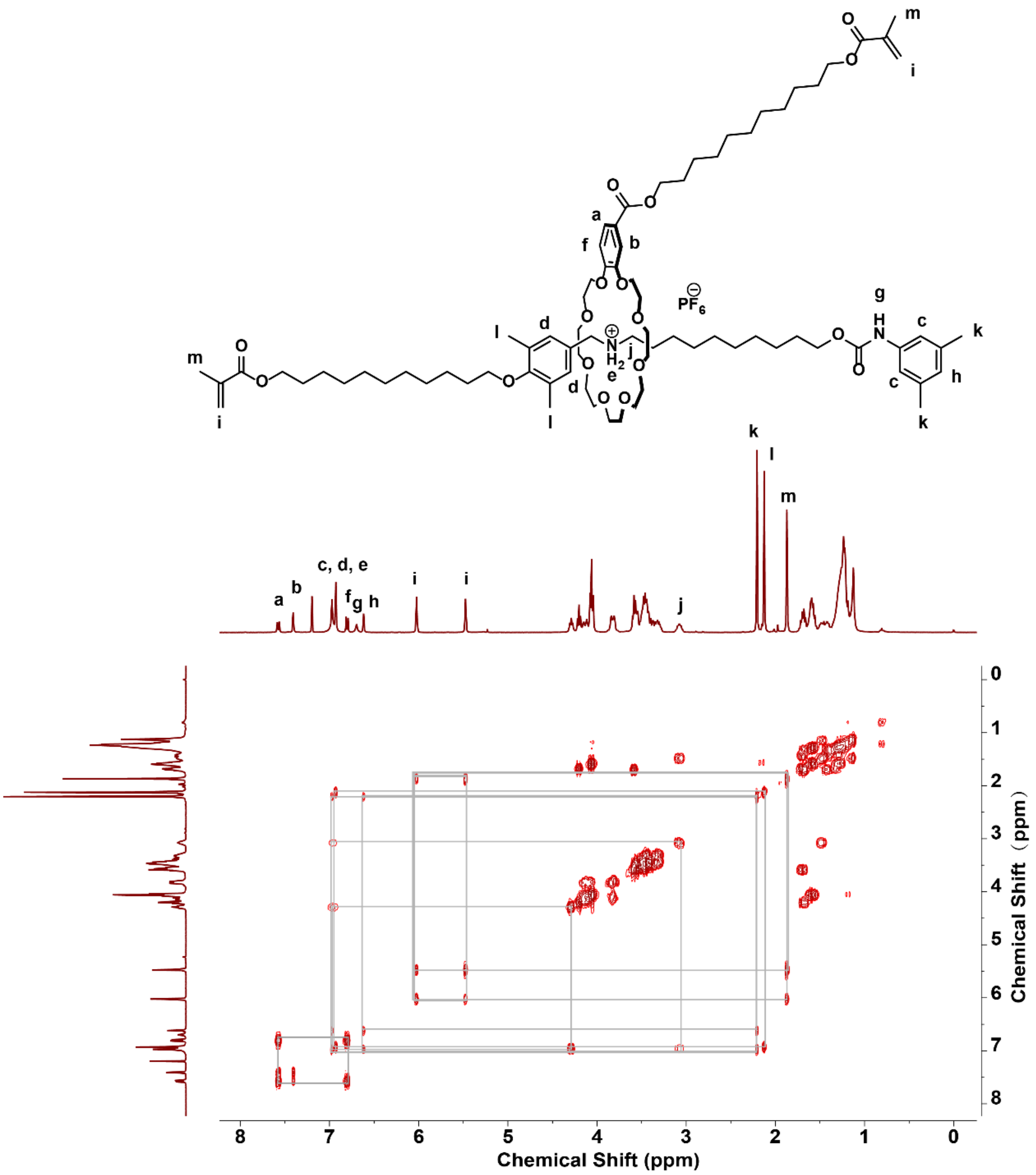

Supplementary Fig. 33 Correlation spectroscopy (COSY) NMR ($CDCl_3$, room temperature, 400 MHz) spectrum of [2]rotaxane.

Synthesis of 2-(3-(6-methyl-4-oxo-1,4-dihydropyrimidin-2-yl)ureido)ethyl methacrylate (10)

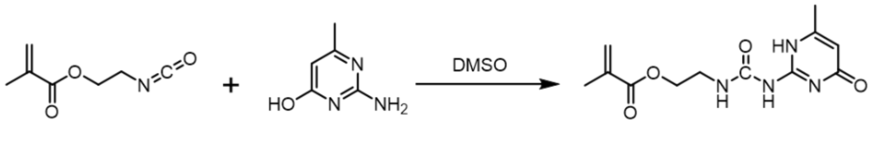

10

2-Amino-4-hydroxy-6-methylpyrimidine (2.00 g, 15.98 mmol) was added into 80 mL of DMSO

and stirred for 10 min at 150 °C. Then the solution was cooled to room temperature and 2-isocyanatoethyl methacrylate (2.70 g, 17.40 mmol) was added into the flask. The mixture was kept stirring for 12 h at room temperature. The white precipitates were collected by filtration, washed with *n*-hexane. Finally, the precipitates were dried under vacuum at 30 °C for 4 h to afford compound 10 as a white powder (2.80 g, 62%). Mp: 195.3–196.1 °C. The ^{1}H NMR spectrum of compound 10 is shown in Supplementary Fig. 34. ^{1}H NMR ($CDCl_3$, room temperature, 400 MHz) δ (ppm): 12.96 (s, 1H), 11.94 (s, 1H), 10.48 (s, 1H), 6.17 (t, J = 1.3 Hz, 1H), 5.78 (s, 1H), 5.60–5.50 (m, 1H), 4.27 (t, J = 5.7 Hz, 2H), 3.55–3.59 (m, 2H), 2.23 (s, 3H), 1.93 (d, J = 1.3 Hz, 3H). The ^{13}C NMR spectrum of compound 10 is shown in Supplementary Fig. 35. ^{13}C NMR ($CDCl_3$, room temperature, 400 MHz) δ (ppm): 172.88, 167.35, 156.82, 154.55, 148.28, 136.16, 125.82, 106.74, 77.34, 77.03, 76.71, 63.10, 41.01, 38.79, 18.97, 18.30. HRESIMS is shown in Supplementary Fig. 36: *m*/*z* calcd for $C_{12}H_{16}N_4O_4$, 281.1244 $[M + H]^+$; found 281.1246 $[M + H]^+$.

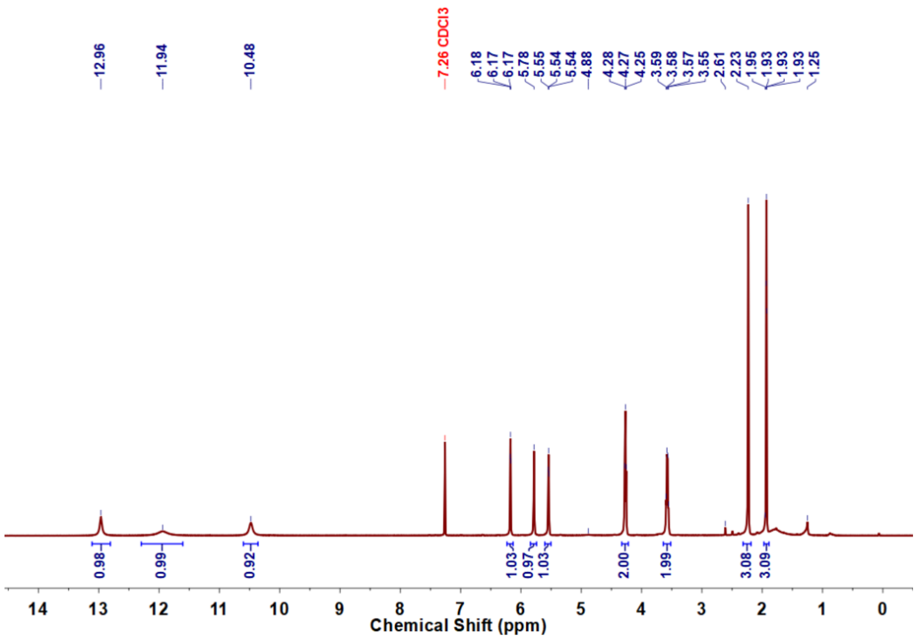

Supplementary Fig. 34 ^{1}H NMR spectrum ($CDCl_3$, room temperature, 400 MHz) of compound 10.

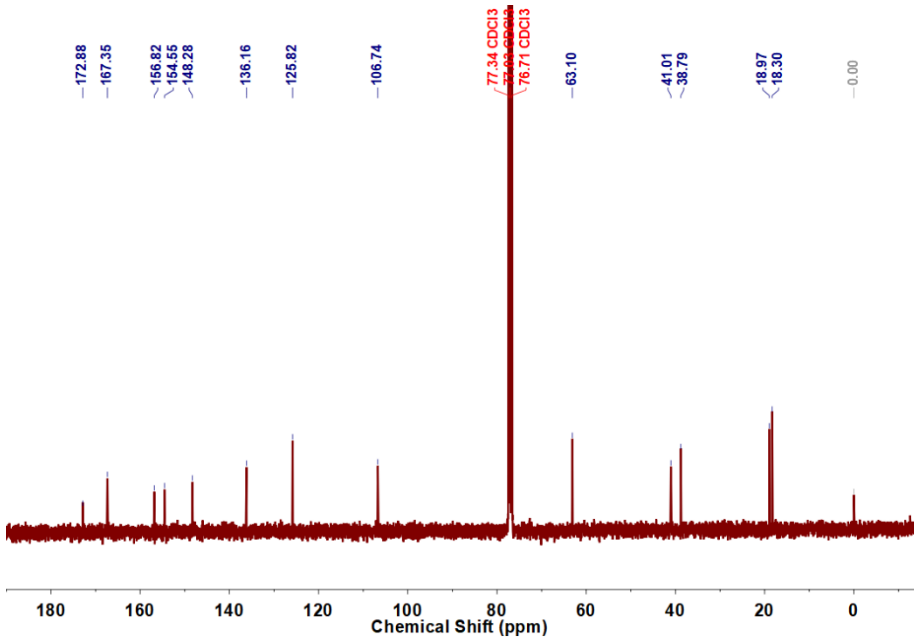

Supplementary Fig. 35 ^{13}C NMR spectrum ($CDCl_3$, room temperature, 400 MHz) of compound 10.

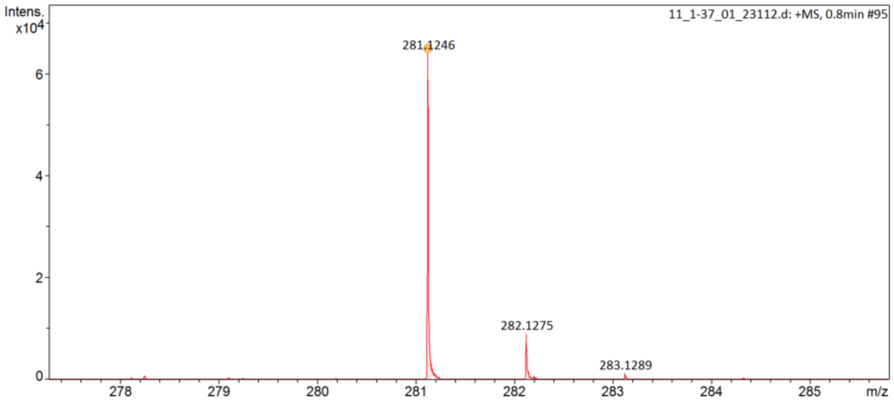

Supplementary Fig. 36 Electrospray ionization mass spectrum of compound 10.

Synthesis of nitrobenzyl (NB)-caged 2-(2-ureido-4[1H]-6-methylpyrimidinone)ethyl methacrylate (NB-UPy)

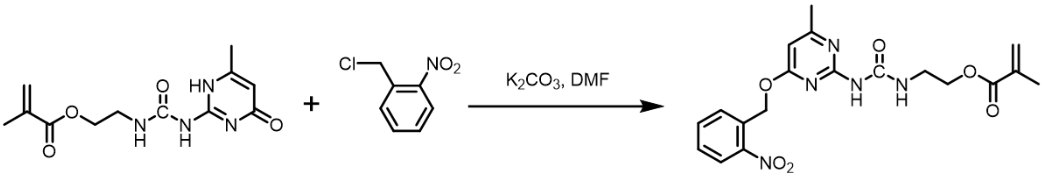

10 **NB-UPy**

2-Nitrobenzyl chloride (3.69 g, 21.50 mmol) was added to a mixture of compound 10 (3.75 g, 13.40 mmol) and K_2CO_3 (2.96 g, 21.50 mmol) in 80 mL of dry DMF. The mixture was stirred at 85 °C for 12 h under N_2. The solution was poured into 200 mL of water and then extracted with ethyl acetate. The organic layer was dried with anhydrous Mg_2SO_4, after which the solvent was removed under reduced pressure and the residue was purified by gel chromatography (CH_2Cl_2/ethyl acetate, 10:1 *v*/*v*) to afford NB-UPy as a yellow solid (3.90 g, 70%). Mp: 127.5–129.3 °C. The ^{1}H NMR spectrum of compound NB-UPy is shown in Supplementary Fig. 37. ^{1}H NMR ($CDCl_3$, room temperature, 400 MHz) δ (ppm): 9.41 (s, 1H), 8.11 (dd, J = 8.1, 1.0 Hz, 1H), 7.69–7.60 (m, 2H), 7.54–7.45 (m, 1H), 7.13 (s, 1H), 6.28 (s, 1H), 6.16–6.11 (m, 1H), 5.72 (s, 2H), 5.55–5.57 (m, 1H), 4.30 (t, J = 5.4 Hz, 2H), 3.67–3.71 (m, 2H), 2.34 (s, 3H), 1.93 (t, J = 1.3 Hz, 3H). The ^{13}C NMR spectrum of NB-UPy is shown in Supplementary Fig. 38. ^{13}C NMR ($CDCl_3$, room temperature, 400 MHz) δ (ppm): 169.63, 167.72, 167.20, 157.16, 154.33, 147.69, 136.16, 133.71, 132.30, 129.09, 128.79, 125.75, 125.02, 100.47, 64.75, 63.84, 38.93, 30.94, 23.71, 18.30. HRESIMS is shown in Supplementary Fig. 39: *m*/*z* calcd for $C_{19}H_{21}N_5O_6$, 438.1384 [M + Na]$^+$; found 438.1383 [M + Na]$^+$.

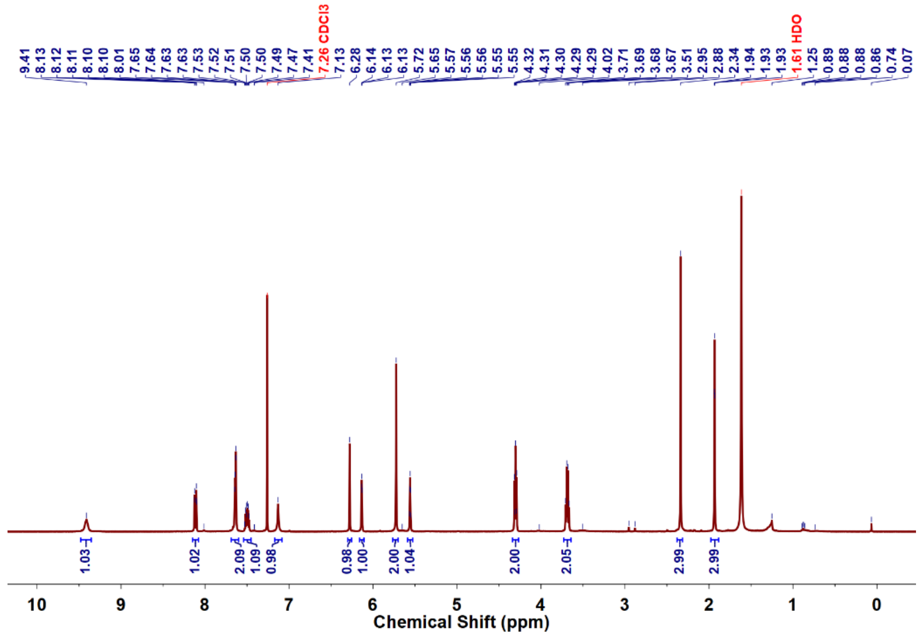

Supplementary Fig. 37 ^{1}H NMR spectrum ($CDCl_3$, room temperature, 400 MHz) of NB-UPy.

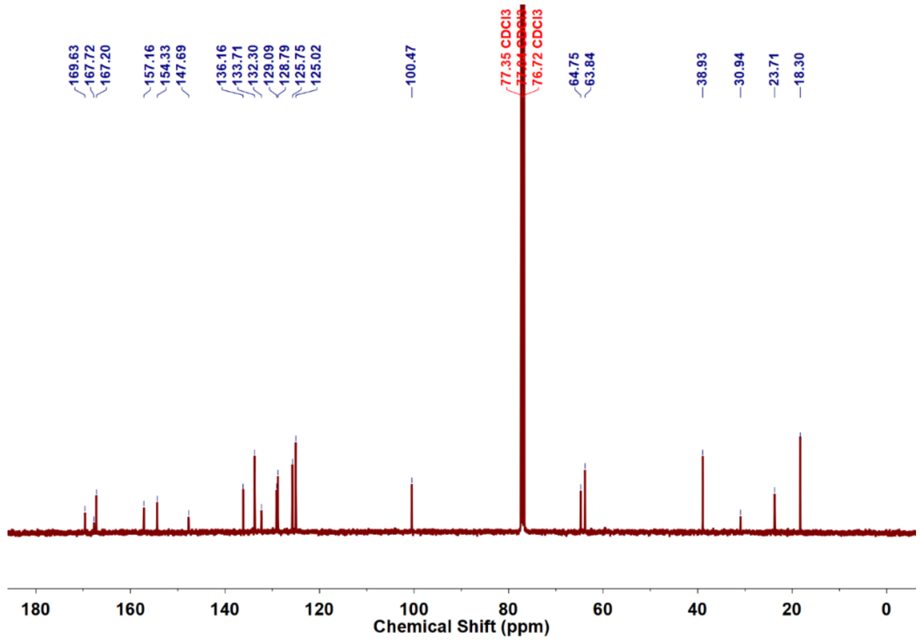

Supplementary Fig. 38 ^{13}C NMR spectrum ($CDCl_3$, room temperature, 400 MHz) of NB-UPy.

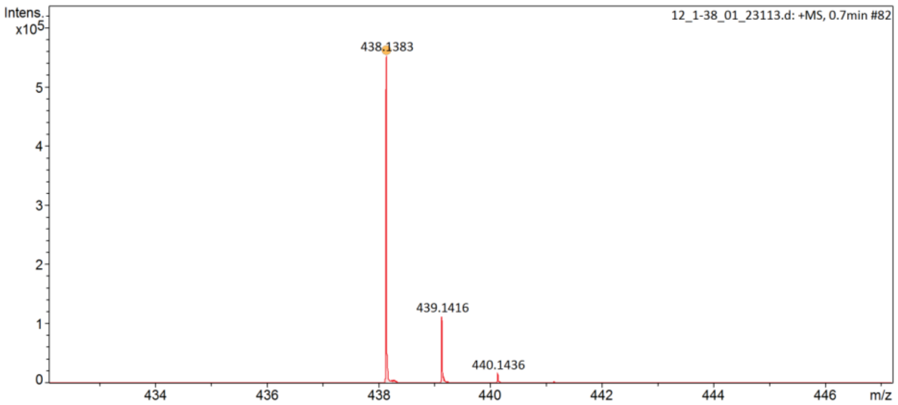

Supplementary Fig. 39 Electrospray ionization mass spectrum of compound NB-UPy.

Synthesis of compound 11

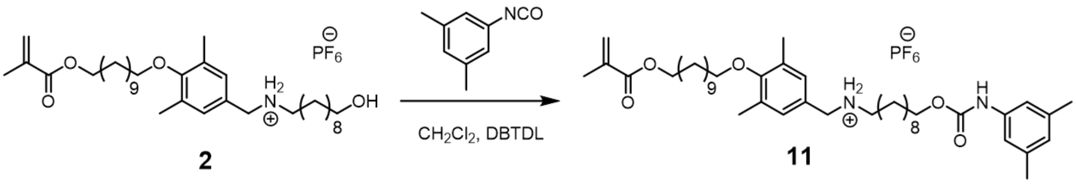

A stirred solution of compounds **2** (0.30 g, 0.43 mmol) and 3,5-dimethylphenyl isocyanate (0.06 g, 0.39 mmol) in CH_2Cl_2 (5 mL) in a round-bottomed flask with a magnetic stir-bar was stirred at room temperature for another 12 h. The solution was evaporated and the residue was purified by gel chromatography (petroleum ether/ethyl acetate, 2:1 *v*/*v*) to afford compound 11 as a yellow waxy solid (0.26 g, 98%). The ^{1}H NMR spectrum of compound 11 is shown in Supplementary Fig. 40. ^{1}H NMR ($CDCl_3$, room temperature, 400 MHz) δ (ppm): 7.03 (s, 2H), 6.98 (s, 2H), 6.70 (s, 1H), 6.60 (s, 1H), 6.09–6.10 (m, 1H), 5.54–5.55 (m, 1H), 4.10–4.14 (m, 4H), 4.04 (s, 2H), 3.72 (t, J = 6.5 Hz, 2H), 2.88 (d, J = 7.8 Hz, 2H), 2.26 (d, J = 3.3 Hz, 12H), 1.94 (t, J = 1.3 Hz, 3H), 1.83–1.73 (m, 2H), 1.70–1.59 (m, 6H), 1.43–1.52 (m, 2H), 1.41–1.22 (m, 28H). The ^{13}C NMR spectrum of compound 11 is shown in Supplementary Fig. 41. ^{13}C NMR ($CDCl_3$, room temperature, 400 MHz) δ (ppm): 167.67, 157.40, 138.75, 136.54, 132.41, 130.20, 125.24, 72.40, 65.36, 64.89, 52.07, 47.42, 30.37, 29.72, 29.54, 29.49, 29.23, 29.02, 28.82, 28.69, 28.61, 26.16, 26.08, 25.98, 25.59, 21.36, 18.34, 16.26. HRESIMS is shown in Supplementary Fig. 42: *m*/*z* calcd for $C_{43}H_{69}N_2O_5$, 693.5201 $[M - PF_6]^+$; found 693.5193 $[M - PF_6]^+$.

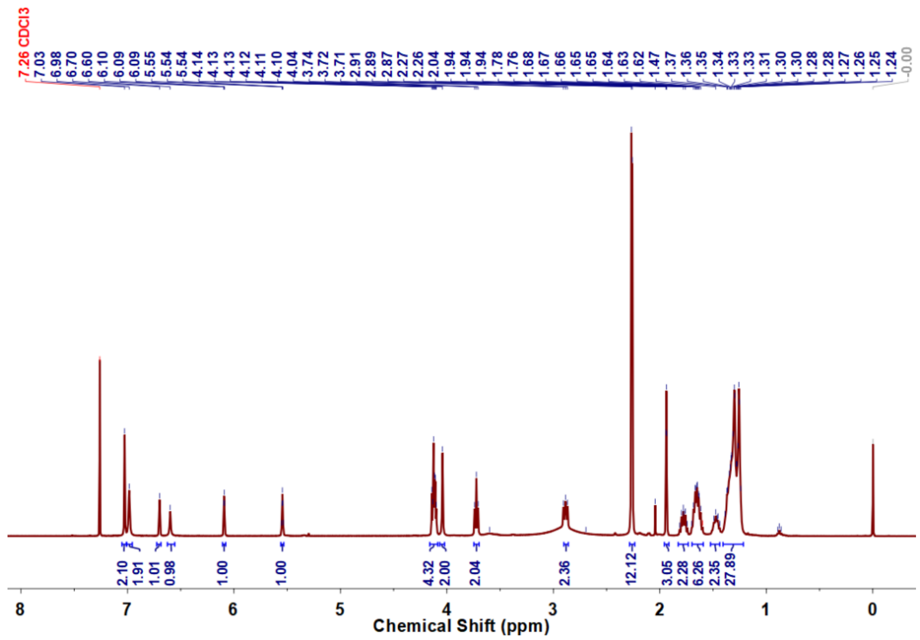

Supplementary Fig. 40 ^{1}H NMR spectrum ($CDCl_3$, room temperature, 400 MHz) of compound 11.

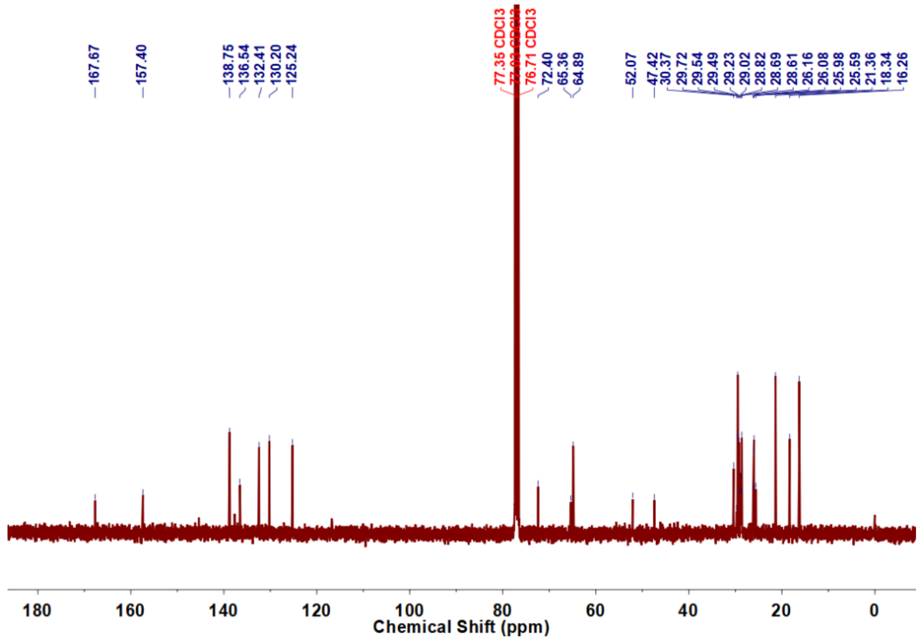

Supplementary Fig. 41 ^{13}C NMR spectrum ($CDCl_3$, room temperature, 400 MHz) of compound 11.

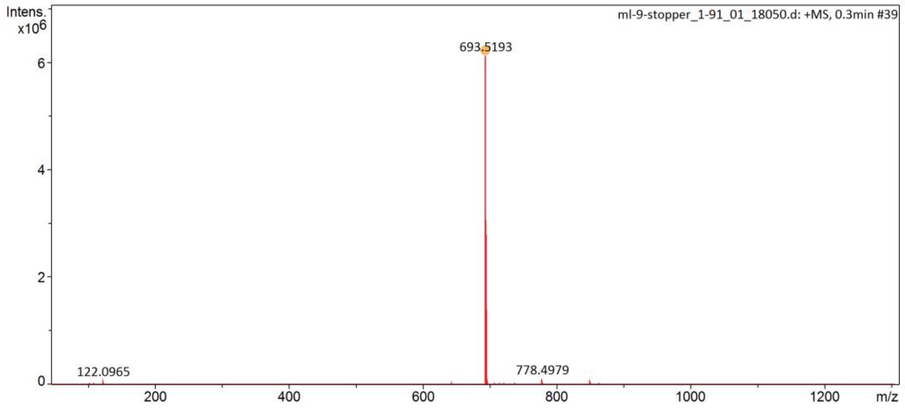

Supplementary Fig. 42 Electrospray ionization mass spectrum of compound 11.

3. Preparation of the MINs and controls.

Supplementary Table 1 The monomer feedings for preparation of MIN-1, 2, 3 and control 1, 2.

Sample	Feeding of monomers			
	[2]rotaxane	NB-UPy	BMA	Molar ratio of [2]rotaxane: NB-UPy
MIN-1	38.00 mg	31.00 mg	1.42 g	1:3
MIN-2	76.00 mg	21.00 mg	1.42 g	1:1
MIN-3	114.00 mg	10.00 mg	1.42 g	3:1
control-1	152.00 mg	-	1.42 g	1:0
control-2	-	42.00 mg	1.42 g	0:1

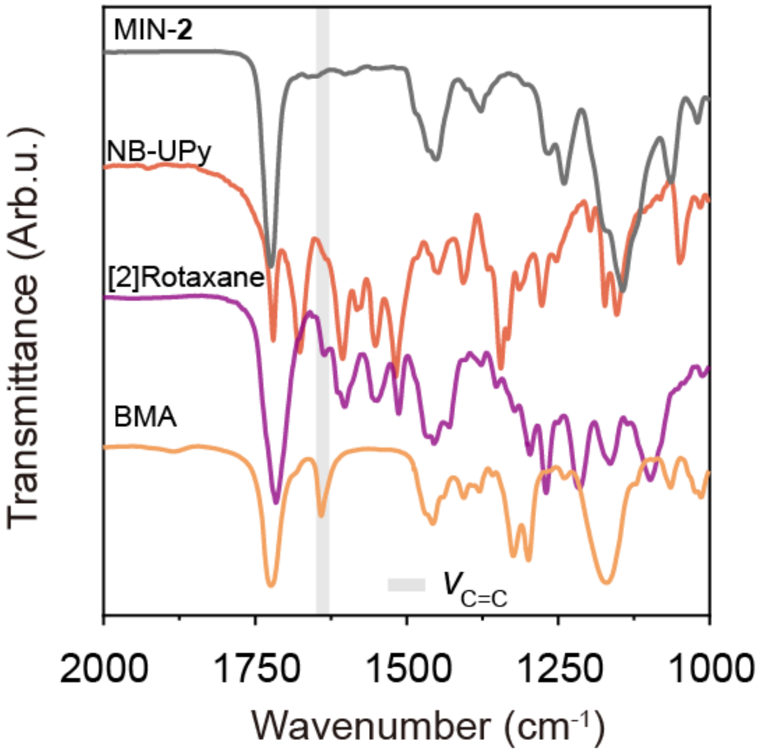

Supplementary Fig. 43 ATR-FT-IR spectra of MIN-2 and the monomers of [2]rotaxane, NB-UPy, and BMA.

4. Preparation of polydimethylsiloxane (PDMS) Substrate

The PDMS elastic sheet was prepared by mixing PDMS prepolymer (Sylgard 184, Dow Corning) in a 10:1 base/curing agent ratio, followed by drop-coating in a Petri dish, degassing in a vacuum oven, and curing at 70 °C for 4 h (thickness approximately 400 μm). Then the sample was cut into 1 cm × 1 cm and 2 cm × 2 cm squares.

5. Monitoring the amplitude (A) and wavelength (λ) of wrinkles

Monitoring the amplitude (A) and wavelength (λ) of responsive wrinkles in real-time:

HCl: The wrinkled pattern was placed in a device filled with hydrogen chloride (HCl) atmosphere. After treatment for different times, the sample was observed by the three-dimensional laser scanning confocal microscopy (LSCM) for real-time data collection.

Et_3N: The wrinkled pattern was placed in a device filled with triethylamine atmosphere. After treatment for different times, the sample was observed by LSCM for real-time data collection.

6. The typical characteristic parameter in the bilayer system

According to linear buckling theory, the classical equation for bending of a stiff film on a more

compliant, elastic substrate is

$$\bar{E}_{\mathrm{f}} I \frac{d^4 z}{dx^4} + F \frac{d^2 z}{dx^2} + kz = 0 \quad \text{(S1)}$$

Here, $\bar{E}$ and F represent the plane-strain modulus and the uniaxially applied force or load, respectively. The subscript f refers to the skin layer in the bilayer system. I and k refer to the moment of inertia and the Winkler's modulus of an elastic half-space, respectively, which are described by Eqs. S2 and S3. The z-axis is defined to be normal to the surface and the x-axis parallel to the direction of F. The sinusoidal vertical deflection of the film (z) is described by Eq. S4.

$$I = \frac{wh^3}{12} \quad \text{(S2)}$$

$$k = \frac{\bar{E}_{\mathrm{s}} w \pi}{\lambda} \quad \text{(S3)}$$

$$z(x) = A \sin \frac{2\pi x}{\lambda} \quad \text{(S4)}$$

where w and h represent the width of the film and its thickness, $\bar{E} = \frac{E}{(1-\nu^2)}$ is the plane-strain moduli, respectively. The subscript s refers to the substrate in the bilayer system, and ν represents the Poisson's ratio.

Eq. S5 is obtained by substituting Eqs. S3 and S4 into Eq. S1.

$$F = 4\bar{E}_{\mathrm{f}} I \left(\frac{\pi}{\lambda}\right)^2 + \frac{\bar{E}_{\mathrm{s}} w}{4} \left(\frac{\pi}{\lambda}\right)^{-1} \quad \text{(S5)}$$

Solving Eq. S5 yields the typical wavelength (λ) and critical force (F_{c}), as given by Eqs. S6 and S7, respectively.

$$\lambda = 2\pi h \left(\frac{\bar{E}_{\mathrm{f}}}{3\bar{E}_{\mathrm{s}}}\right)^{1/3} \quad \text{(S6)}$$

$$F_{\mathrm{c}} = \frac{\bar{E}_{\mathrm{f}} wh}{4} \left(\frac{3\bar{E}_{\mathrm{s}}}{\bar{E}_{\mathrm{f}}}\right)^{2/3} \quad \text{(S7)}$$

The critical strain (ε_{c}) is obtained from Eq. S7 and given as Eq. S8.

$$\varepsilon_{\mathrm{c}} = \frac{\sigma_{\mathrm{c}}}{\bar{E}_{\mathrm{f}}} = \frac{F_{\mathrm{c}}}{hw\bar{E}_{\mathrm{f}}} = \frac{1}{4} \left(\frac{3\bar{E}_{\mathrm{s}}}{\bar{E}_{\mathrm{f}}}\right)^{2/3} \quad \text{(S8)}$$

The amplitude (A) can be calculated from geometric deformation, as shown in Eq. S9.

$$\varepsilon - \varepsilon_{\mathrm{c}} = \frac{1}{\lambda} \int_0^{\lambda} \sqrt{1 + \left(\frac{dz}{dx}\right)^2}\, dx - 1 \approx \frac{\pi^2 A^2}{\lambda^2} \quad \text{(S9)}$$

where $\varepsilon = (\alpha_{\mathrm{s}} - \alpha_{\mathrm{f}}) \times \Delta T$ is the thermoinduced compressive stress. Solving Eq. S9 yields the typical amplitude, as given by Equation S10.

$$A = h \sqrt{\frac{\varepsilon - \varepsilon_{\mathrm{c}}}{\varepsilon_{\mathrm{c}}}} \quad \text{(S10)}$$

In our experiments, $\alpha_s \geq \alpha_f$, $\alpha_s \approx 300 \times 10^{-6}$/°C, and $\Delta T \approx 75$°C; therefore, $\varepsilon \approx 2.25\%$. On the other hand, the Poisson's ratios of the film and substrate are $\nu_f = 0.4$ and $\nu_s = 0.5$; the thickness of top film is $h_f \approx 1.2$ μm $E_f = 452$ MPa and $E_s = 2$ MPa. The calculated value of the wrinkle wavelength by Eq. S6 is $\lambda \approx$33.07 μm.

7. Thermal properties characterization of the MINs and controls.

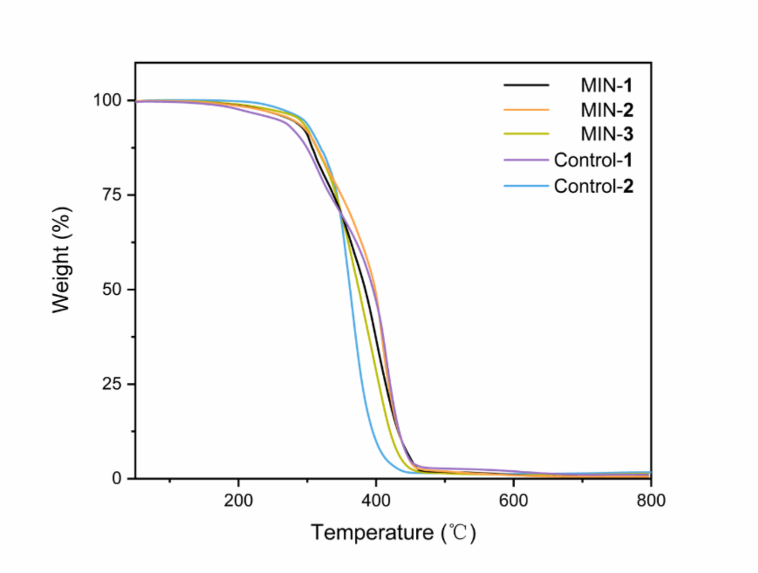

Supplementary Fig. 44 TGA curves of the MINs and controls recorded under N_2 flow (50 mL/min) with a heating rate of 20 °C/min.

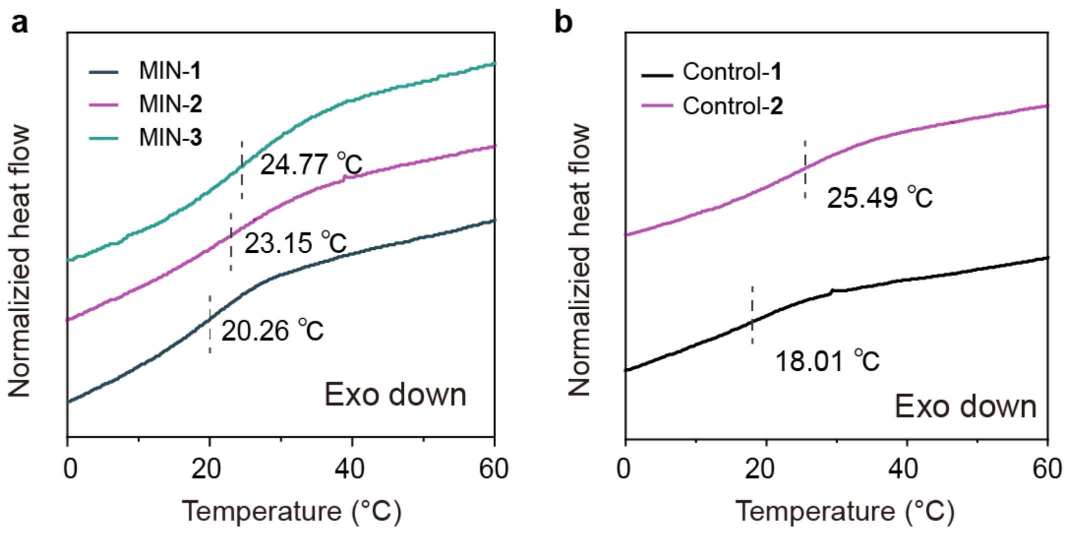

Supplementary Fig. 45 DSC curves of (a) MINs and (b)controls recorded in the second heating scan from 0 to 60 °C with a heating rate of 20 °C /min.

8. Mechanical Properties of the MIN-2.

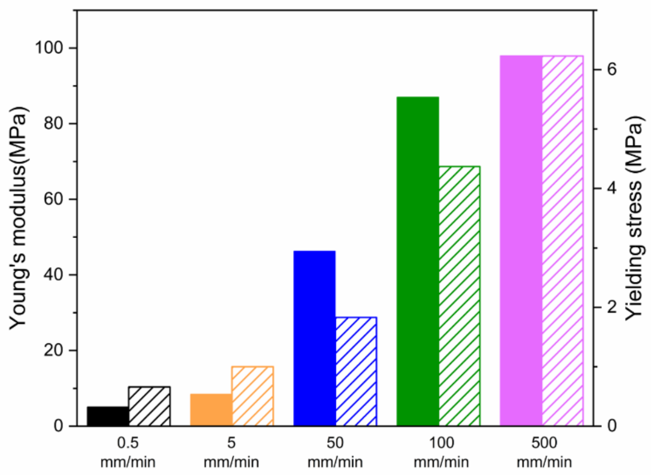

Supplementary Fig. 46 Young's modulus and yielding stress of MIN-2 obtained from the tensile stress–strain curves at different stretching rates.

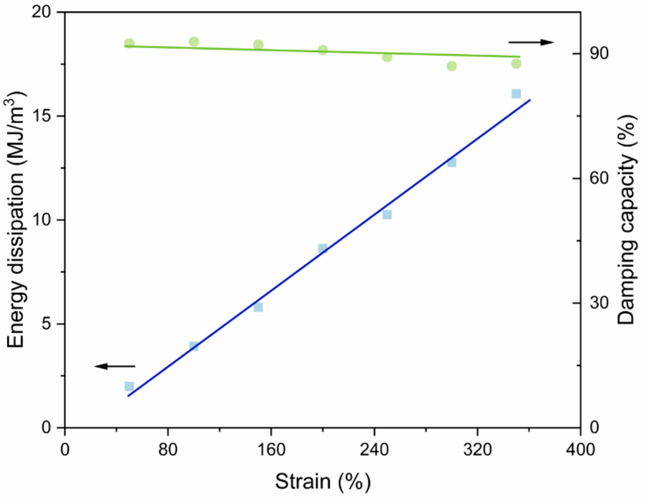

Supplementary Fig. 47 Energy dissipation and damping capacity for each circle of the cyclic tensile test curves.

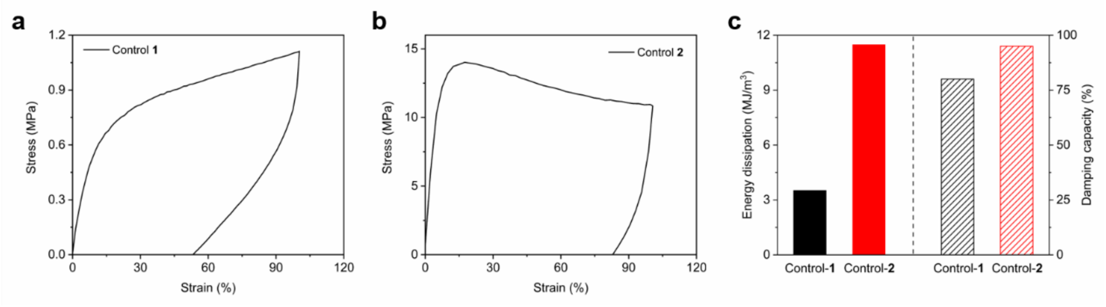

Supplementary Fig. 48 Cyclic tensile test curves of (a) control-1 and (b) control-2 under an applied strain of 100% with a deformation rate of 100 mm/min at room temperature. (c) Energy dissipation and damping capacity of control-1 and control-2 calculated from their cyclic tensile test curves.

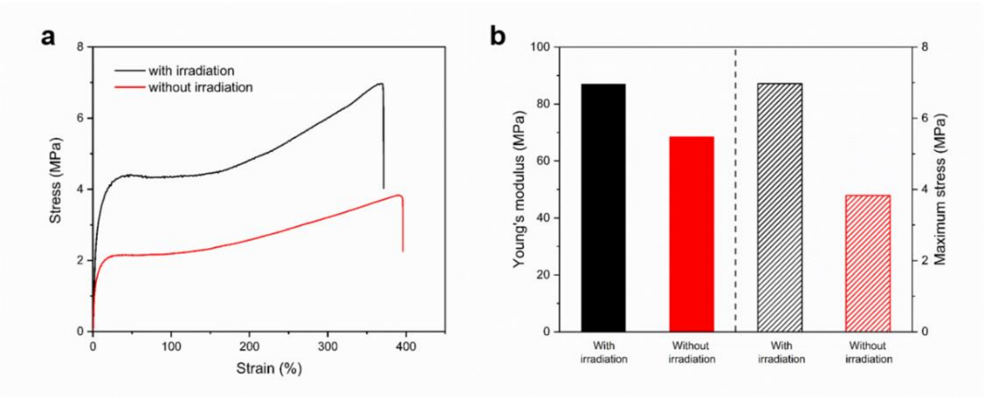

Supplementary Fig. 49 (a) Stress–strain curves of MIN-2 with and without irradiation recorded with a deformation rate of 100 mm/min. (b) Young's moduli and maximum stress calculated based on the stress–strain curves of MIN-2 with and without irradiation.

9. Tunable wrinkles and light gratings based on MINs/PDMS bilayer.

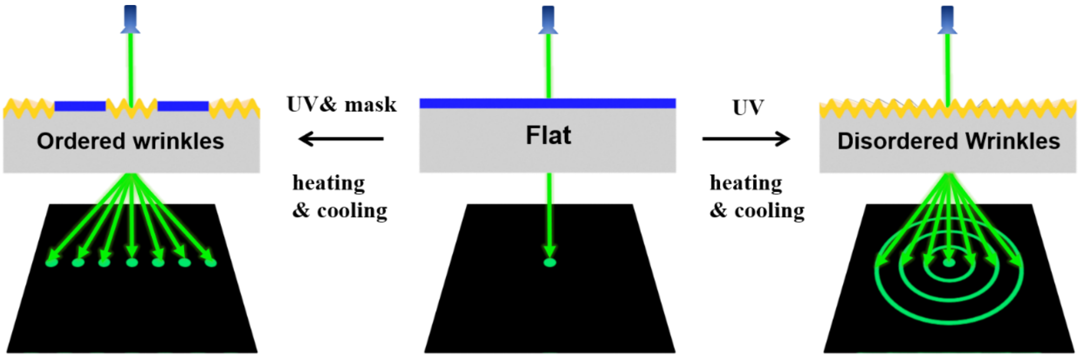

Supplementary Fig. 50 Schematic illustration of the light diffraction patterns based on the wrinkles.

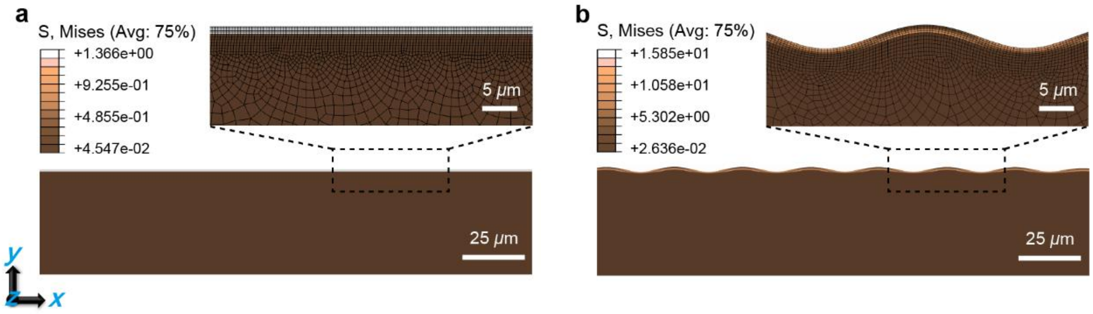

Supplementary Fig. 51 Finite element simulation[3] of the stress distributions of the (a) unexposed and (b) exposed MIN/PDMS bilayers after being heating treatment.

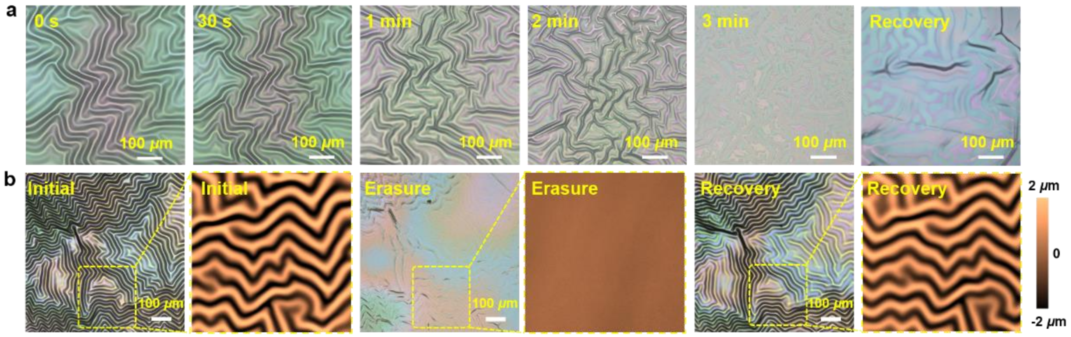

Supplementary Fig. 52 (a) 2D LSCM images of erasure/recovery process of the wrinkling patterns in the atmosphere containing HCl for different time (0, 30 s, 1 min, 2 min, 3 min), and after heating/ cooling treatment. The partial pressure of HCl was approximately 90 Pa. Scale bars: 100 μm. (b) LSCM images of the initial and erasure of wrinkling patterns after the sample being exposed to the atmosphere containing Et_3N for 15 min and recovery 2D LSCM images after heating and cooling.

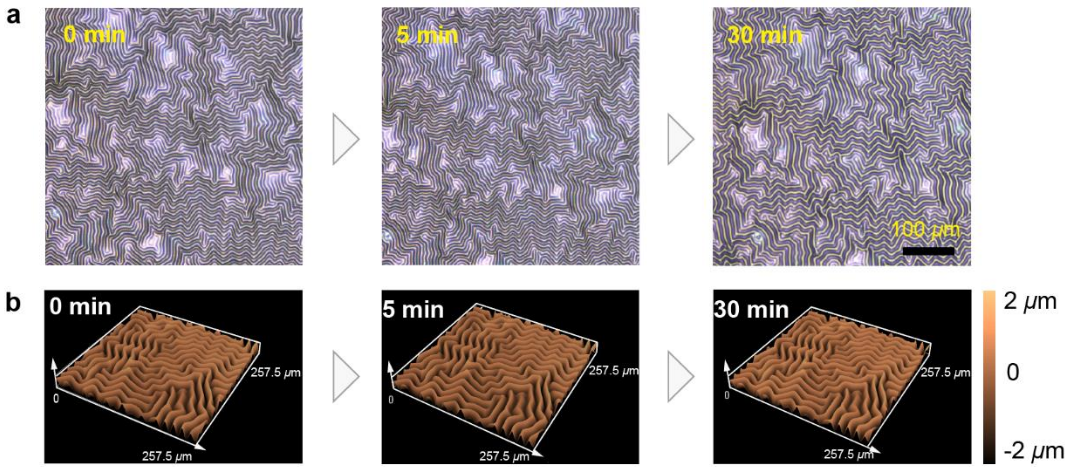

Supplementary Fig. 53 (a) 2D LSCM images of wrinkles of the control sample in the atmosphere containing Et_3N for different time: 0, 5, 30 min, respectively. Scale bar: 100 μm. (b) 3D LSCM images of wrinkles with time at room temperature after the control sample being exposed to the atmosphere containing Et_3N for 15 min. Evolution of the erased wrinkle induced by stress relaxation using finite element modeling.

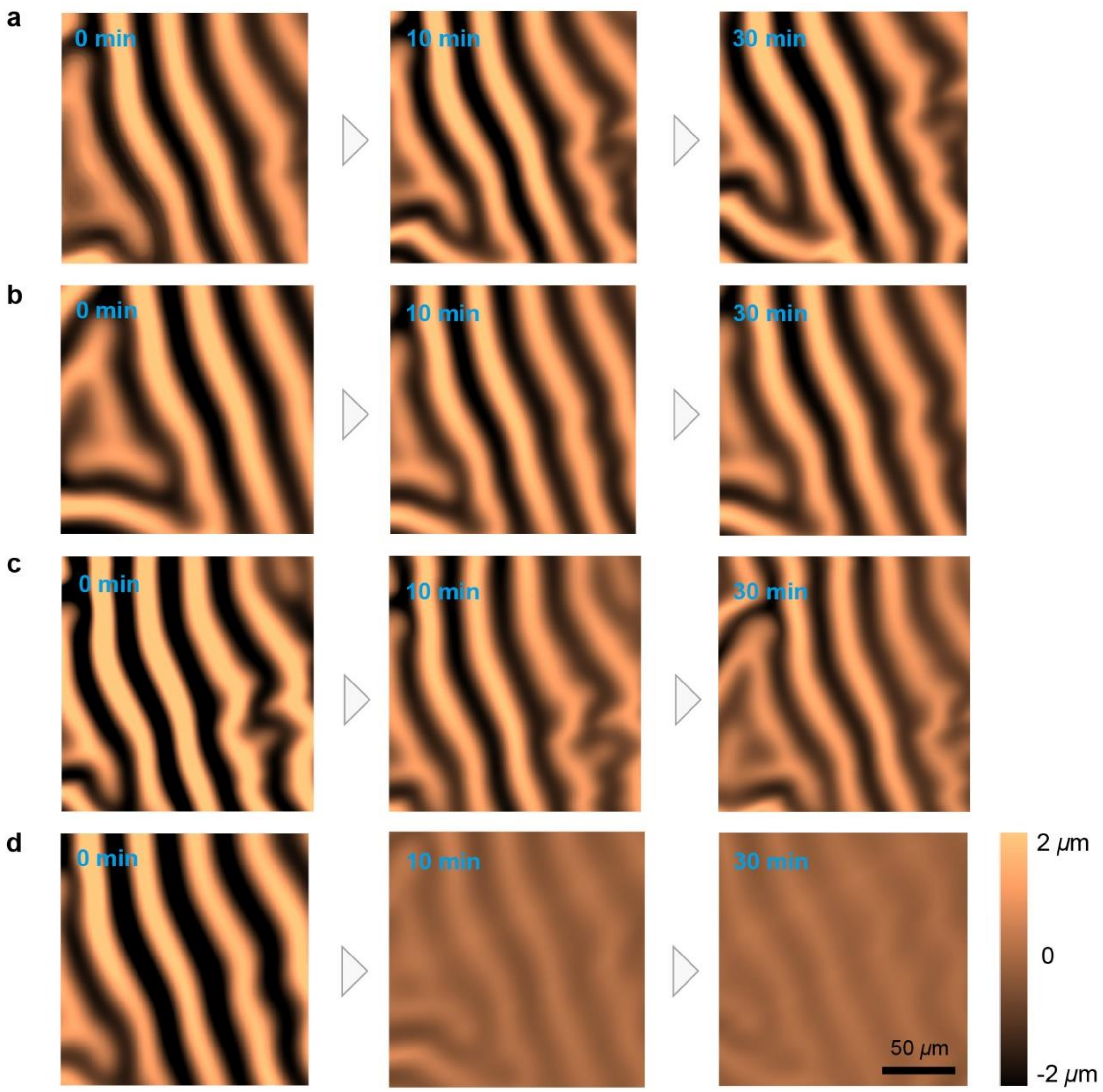

Supplementary Fig. 54 LSCM images of erasure process of wrinkling patterns after the sample being exposed to the atmosphere containing Et_3N for (a) 0 min, (b) 3 min, (c) 7 min and (d) 15 min. Scale bar: 50 μm.

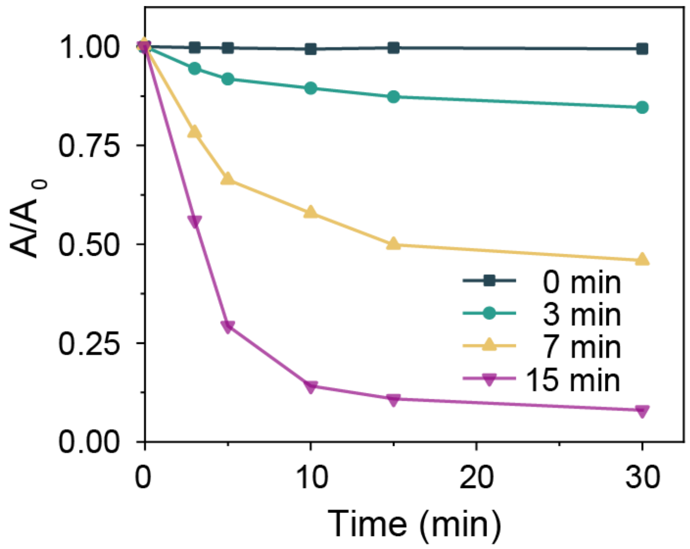

Supplementary Fig. 55 The normalized amplitude of the wrinkles as a function of the time after the sample being exposed to the atmosphere containing Et_3N for 0 min, 3 min, 7 min and 15 min.

10. Finite element (FE) simulations of the generation and erasure process of dynamic wrinkles

As shown in Supplementary Fig. 56, FE analysis of the stress distribution and the evolution process of the presented bilayer before and after 365 nm UV irradiation was performed in the static environment of the Abaqus software (version 2020)[3]. The MINs and soft PDMS bilayer system was constructed from top to bottom according to the experiment condition, and their interfaces were assumed to be bonded without slippage. As for the boundary conditions, the bottom of the PDMS elastic substrate was constrained in the *y* direction, and the PDMS could move only along the indentation direction when subjected to a linearly increasing displacement load. The linear elastic model was utilized to qualitatively describe the constitutive behavior of the wrinkling materials.

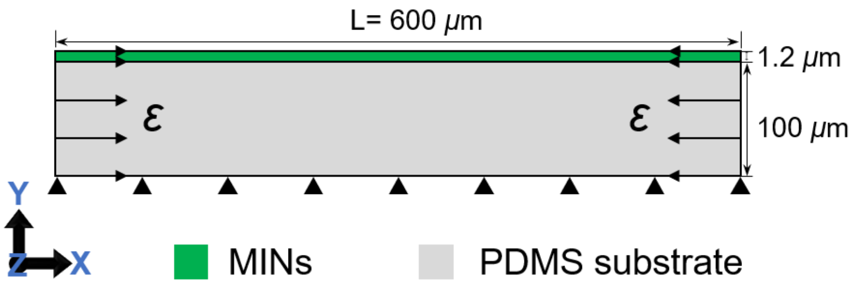

Supplementary Fig. 56 Schematic illustration of the 2D cross-section in *xy*-plane for the finite element model.

To investigate insightful theoretical mechanism of the adjustable wrinkle structures, finite element (FE) simulations for the dynamic erasable wrinkles based on the responsive [2]rotaxane-induced stress relaxation post Et_3N treatment were conducted by using the Abaqus software. As shown in Supplementary Fig. 57, the FE-simulated A and λ of wrinkles are well consistent with the experiment values obtained by LSCM. Since the stress relaxation is triggered by the Et_3N-induced movement of [2]rotaxane motifs, the characteristic A of multi-sensitive wrinkles decreases significantly and the top stiff surface gradually becomes flat, which suggests a novel regulatory mechanism in the presented system.

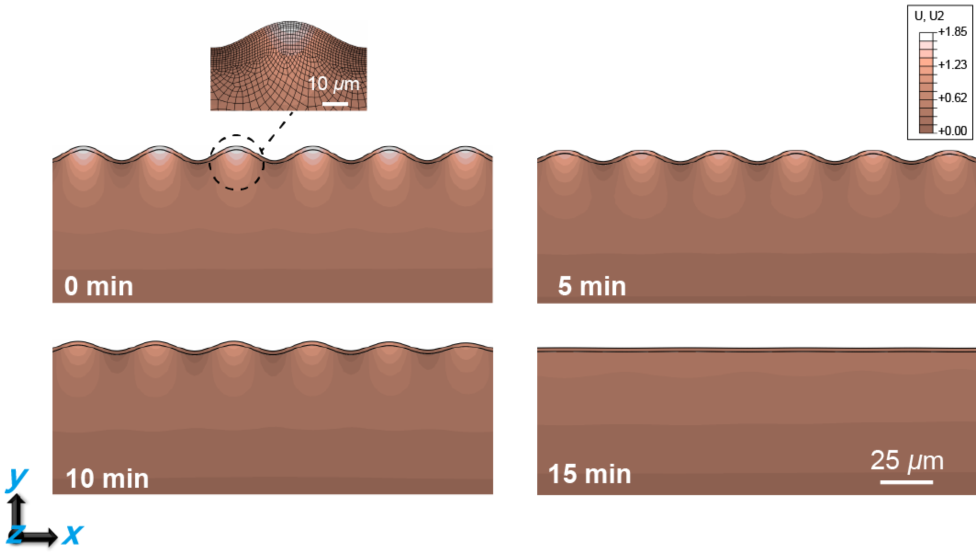

Supplementary Fig. 57 Evolution of the erased wrinkle induced by stress relaxation using finite element modeling.

11. FE simulation to predict the compared evolution of wrinkles and strain

In order to investigate the different evolution process of the dynamic wrinkles upon HCl and Et_3N treatment, FE simulation is utilized to simulate the wrinkling, flatten, and re-wrinkling cycle using a commercial software package Abaqus. The thickness of the functional film is 1.2 μm, and the thickness of substrate is 45 μm which is 37.5 times thicker than the film. The modelled area is a 100 × 100 μm square and is meshed to solid elements. The compliant substrate with a Young's modulus of 2 MPa is modelled as solid element C3D4, and the thin film with a Young's modulus of 425 MPa is modelled as solid element C3D8R. The Poisson's ratio for both substrate and film are set to be 0.45. The density of both substrate and film is set to be 1e^{-13} to minimize the kinetic energy.

The thermal expansion of substrate and film is set to be $3e^{-3}$/°C and $1e^{-4}$/°C. The quasi-static analysis was conducted with Abaqus Dynamic Explicit Module for better convergence. In order to trigger the surface instability and simulate the differences of the two response mechanisms, a random imperfection of the thin film was introduced into the model. The randomly picked imperfection is unchanged during the wrinkling disappearing/regeneration process upon Et_3N treatment, due to the stability of crosslinked MINs network. As compared, the randomly picked imperfection is changed after HCl treatment due to the dissipating energy through network rearrangement. To initially generate a wrinkling pattern, a uniform isotropic decreased temperature of 30 °C across the bilayer model was introduced, which corresponds to about 8.7% compressive thermal strain on film. To simulate the released wrinkling process in response to HCl or Et_3N treatment, a decreased compressive strain is applied. Then, the temperature field is again decreased with 30 °C to fully re-generate the wrinkles, showing the different recovery process of wrinkles.

Supplementary References:

1 Chen, Y. & Baker, G. L. Synthesis and properties of ABA amphiphiles. *J. Org. Chem.* **64**, 6870–6873 (1999).

2 Wang, Y. *et al.* Mechanically interlocked [an]daisy chain networks. *Chem* **9**, 2206–2221 (2023).

3 Xie, M. *et al.* Pattern memory surface (PMS) with dynamic wrinkles for unclonable anticounterfeiting. *ACS Mater. Lett.* **1**, 77–82 (2019).
